# Plants of the Genus *Terminalia*: An Insight on Its Biological Potentials, Pre-Clinical and Clinical Studies

**DOI:** 10.3389/fphar.2020.561248

**Published:** 2020-10-08

**Authors:** Gitishree Das, Do-Yeong Kim, Chen Fan, Erick P. Gutiérrez-Grijalva, J. Basilio Heredia, Veeranoot Nissapatorn, Watcharapong Mitsuwan, Maria Lourdes Pereira, Muhammad Nawaz, Abolghasem Siyadatpanah, Roghayeh Norouzi, Barbara Sawicka, Han-Seung Shin, Jayanta Kumar Patra

**Affiliations:** ^1^ Research Institute of Biotechnology & Medical Converged Science, Dongguk University-Seoul, Goyangsi, South Korea; ^2^ Skin Research Institute of Singapore, Agency for Science, Technology and Research, A∗STAR, Singapore, Singapore; ^3^ Laboratorio de Alimentos Funcionales y Nutracéuticos, Cátedras CONACYT–Centro de Investigación en Alimentación y Desarrollo, Culiacán, México; ^4^ Laboratorio de Alimentos Funcionales y Nutracéuticos, Centro de Investigación en Alimentación y Desarrollo, Culiacán, México; ^5^ School of Allied Health Sciences, Research Excellence Center for Innovation and Health Products (RECIHP) and World Union for Herbal Drugs Discovery (WUHeDD), Walailak University, Nakhon Si Thammarat, Thailand; ^6^ CICECO-Aveiro Institute of Materials and Department of Medical Sciences, University of Aveiro, Aveiro, Portugal; ^7^ Department of Nano-Medicine Research, Institute for Research and Medical Consultations (IRMC), Imam Abdulrahman Bin Faisal University, Dammam, Saudi Arabia; ^8^ Ferdows School of Paramedical and Health, Birjand University of Medical Sciences, Birjand, Iran; ^9^ Department of Pathobiology, Faculty of Veterinary Medicine, University of Tabriz, Tabriz, Iran; ^10^ Faculty of Agrobioengineering, Department of Plant Production Technology and Commodities Science, University of Life Sciences in Lublin, Lublin, Poland; ^11^ Department of Food Science & Biotechnology, Dongguk University-Seoul, Goyangsi, South Korea

**Keywords:** antiviral, biological activities, clinical studies, phytogeography, pneumonia, *Terminalia* sp.

## Abstract

The evaluation and confirmation of healing properties of several plant species of genus *Terminalia* based on their traditional uses and the clinical claims are of utmost importance. Genus *Terminalia* has received more attention to assess and validate the therapeutic potential and clinical approval due to its immense folk medicinal and traditional applications. Various species of *Terminalia* genus are used in the form of herbal medicine and formulations, in treatment of diseases, including headache, fever, pneumonia, flu, geriatric, cancer, to improve memory, abdominal and back pain, cough and cold, conjunctivitis, diarrhea, heart disorder, leprosy, sexually transmitted diseases, and urinary tract disorders. These are reported to possess numerous biological properties, counting: antibacterial, antifungal, antiinflammatory, antiviral, antiretroviral, antioxidant, and antipa7rasitic. This current research review aims to update the detailed biological activities, pre-clinical and clinical studies of various extracts and secondary metabolites from several plant species under the genus *Terminalia*, along with information on the traditional uses and chemical composition to develop a promising strategy for their potential applications in the form of medicine or use in modern drug formulations for treating diseases like pneumonia, flu, and other types of viral infections or controlling human contagions.

## Introduction

Natural products in medicinal plants are essential sources for drug discovery (Harvey et al., 2015). It has been reported that natural products take up to 35% of the global medicine market, which is approximately 385 billion US dollars (Calixto, 2019). To discover the medical values of natural products, it is critically important to understand the ethnopharmacological uses of various medicinal plants, as it provides reliable information on the evaluation of natural products existing in those medicinal plants (Buenz et al., 2018). Although the development of modern medicines is quickly growing, there is still a large amount of population preferring herbal medicines than the conventional system of medicines due to their effectiveness, lack of medical alternatives, enhancing cost of modern medicines, and cultural preferences (Heinrich, 2000; Tabuti et al., 2003; Amalraj and Gopi, 2017). Based on the data from WHO, about 80% of the global population depends on traditional medicine, and 60% of the Indian population in rural areas use herbal medicines (Amalraj and Gopi, 2017). These natural medicines are generally easy to access, safe, cost-effective, and efficient (Amalraj and Gopi, 2017). Except for the medical values, various plants are also widely used as food (Konczak et al., 2014), health care products (Kim and Song, 2013), veterinary medicine (Upadhyay et al., 2011), possessing extensive impacts on daily life.

**Figure 1:**
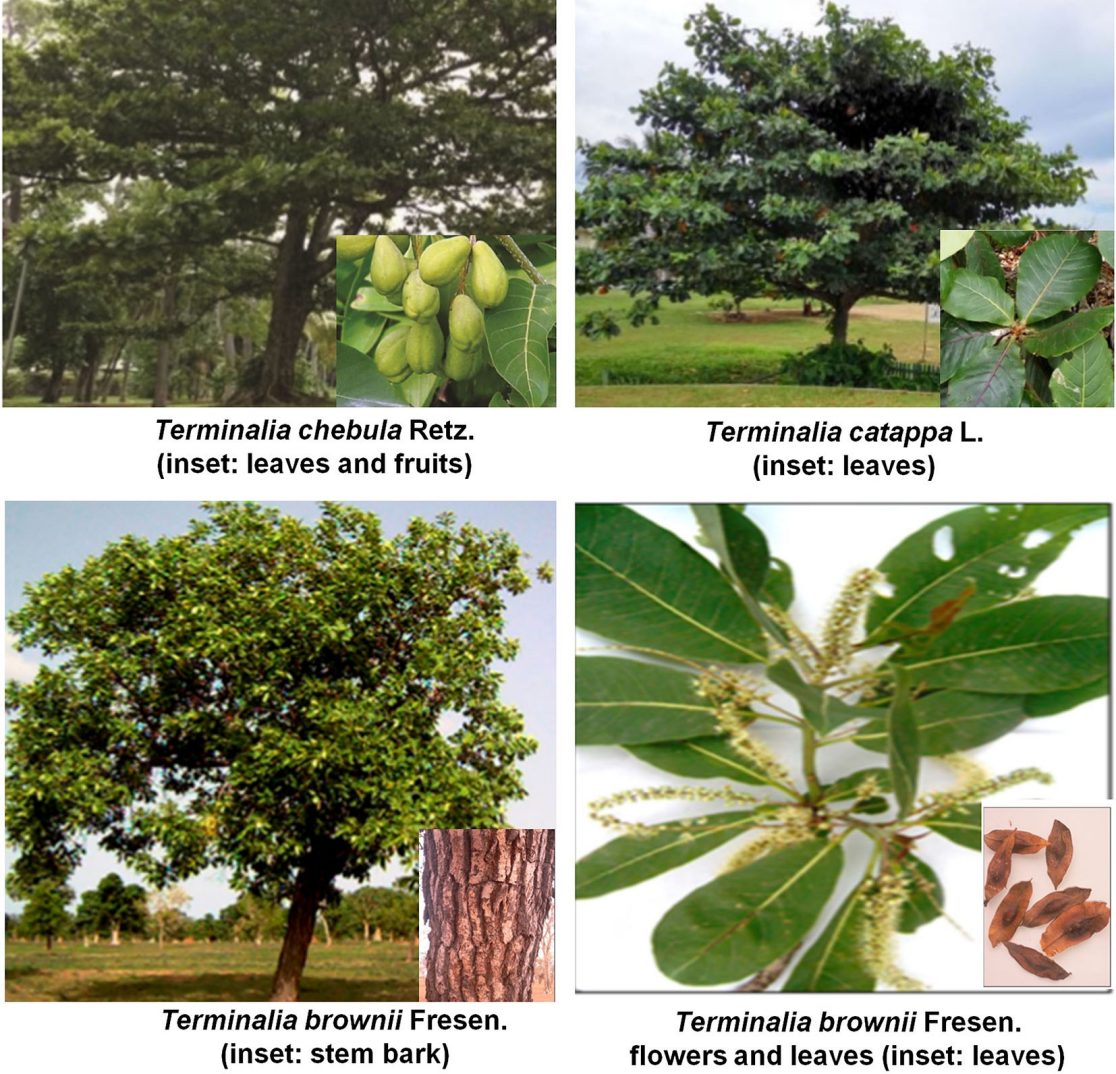
Some of the common *Terminalia* sp. Reproduced under the Creative Commons Attribution License (Afshari et al., 2016; Marjenah and Putri, 2017; Salih et al., 2017).

*Terminalia* sp., family *Combretaceae*, is distributed worldwide, with around 250 species, especially in South Asia, Australia, and South Africa. Among them, more than 50 species are used as food (Fan et al., 2015). A list of some of the important plant species under *Terminalia* genus with medicinal potential is provided in **Supplementary Table 1**, and a few of the species are shown in **Figure 1**. These are some of the most widely used medicinal plants in the global ethnopharmacology such as traditional Chinese, Tibetan, and Indian Ayurvedic medicine system. For instance, fruits of *Terminalia ferdinandiana* Exell, are rich in (1) vitamin C and thereby being consumed as food in Australia (Konczak et al., 2014). Several *Terminalia* species exhibit nutraceutical value with numerous health benefits, including the treatment of some diseases (Cock, 2015). For example, fruits of *T. bellirica* (Gaertn.) Roxb. and *T. chebula* Retz. usually form Triphala, the well-known polyherbal formulation in Ayurvedic and Thai folk medicine, due to its pharmacological applications as a laxative, detoxifying, and rejuvenating effects (Intharuksa et al., 2016). Phytochemical studies in 39 species led to the identification of 368 compounds, including terpenoids, tannins, flavonoids, phenylpropanoids, simple phenolics, among others (Zhang et al., 2019). Some of these compounds demonstrated different bioactivities that were explored through *in vitro* or *in vivo* assays. Of note, among these properties, antidiabetic and antiobesity, anticancer, antiinflammatory, antimicrobial, antimalarial, antioxidant, antitumor have been reported in several plant species. It has been documented that *T. arjuna* (Roxb. ex DC.) Wight & Arn. is traditionally used for cardioprotective and hepatoprotective purposes in India and Sri Lanka (Kapoor et al., 2014); *T. bellirica* (Gaertn.) Roxb., is widely used on treating diarrhea (Pandey et al., 2017); *Terminalia brownii* Fresen., has been used to treat brown-erythematous excoriated papules and plaques (Kibar Ozturk et al., 2018); *T. chebula* Retz. is widely used to treat dementia, constipation, and diabetes in traditional Indian and Iranian medicine (Jokar et al., 2016), etc. Therefore, the active compounds existing in *Terminalia* sp. have great potential applications in various diseases. This review presents the much-needed update on the folk medicinal uses, phytochemistry, chemical composition, and pharmacological applications of numerous plants of genus *Terminalia*, along with information on the pre-clinical and clinical trials of their compounds. We aim to improve the understanding of the mechanisms underlying the medical use of *Terminalia* sp., stimulating the use of *Terminalia* sp. in modern drug discovery.

## Research Methodology

To identify information on the biological potential, pre-clinical, and clinical studies of *Terminalia* sp. this review compiled information from recent literature (2010–2020) from the Scopus, Web of Science, and PubMed databases. The keywords used for the literature research included the terms: *Terminalia*, antioxidant, cancer, diabetes, antidiabetic, antiobesity, inflammation, antiinflammatory, antimicrobial, antifungal, antiparasitic, nanoparticles, and *in vivo* studies.

## Updates on the Research on *Terminalia* sp. Till Date

**Figure 2:**
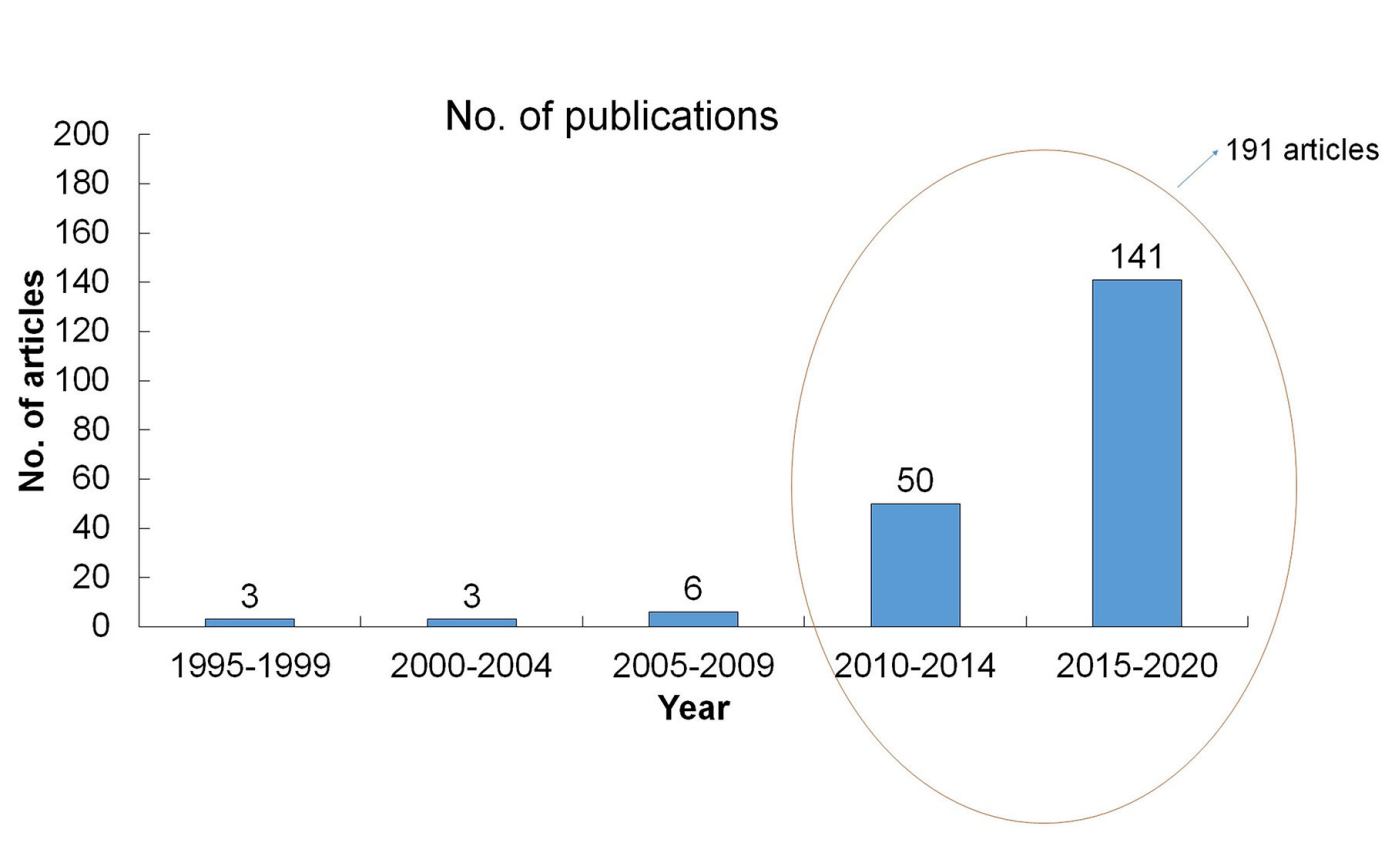
A detailed list of publications on *Terminalia* sp. Till date. The information was collected from PubMed (https://pubmed.ncbi.nlm.nih.gov/?term=Terminalia+sp.&filter=ds1.y_10, 15 July 2020).

It has been known since ancient days that medicinal plants are sources of bioactive compounds. As per the PubMed database, a total of, 201 articles were published on the *Terminalia* sp., out of which around 191 articles were published during the year 2010–2020 (**Figure 2**). Among these articles, maximum was available as full texts whereas only three articles were reviews and one article is on the clinical trial.

**Figure 3:**
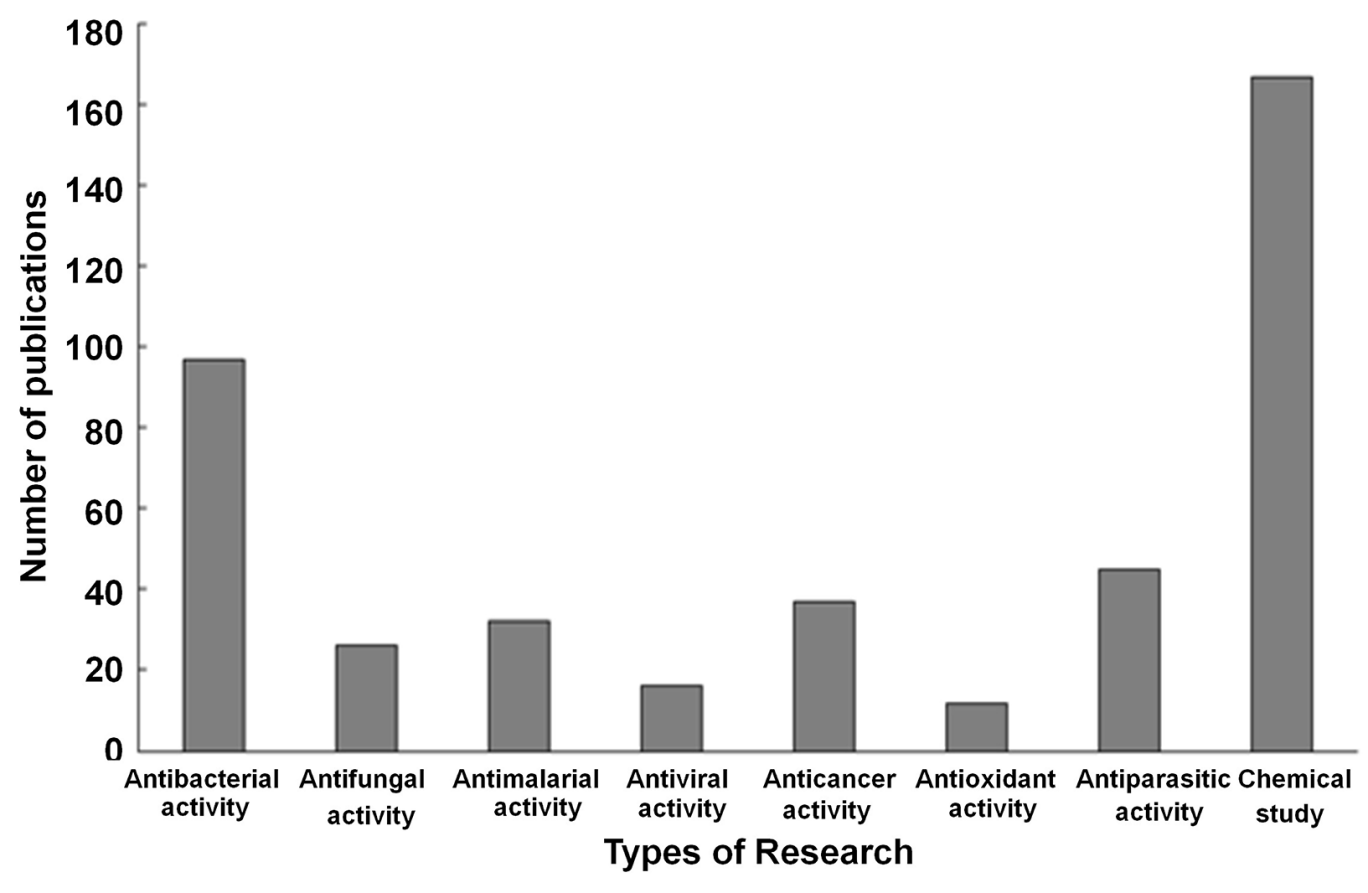
Trends in *Terminalia* sp. research within 2010-2020. The information was collected from PubMed (https://pubmed.ncbi.nlm.nih.gov/?term=Terminalia+sp.&filter=ds1.y_10, 15 July 2020).

Based on the objectives of the current review, the present article focuses on *Terminalia* sp., belonging to the family *Combretaceae*, possesses various bioactive properties including antibacterial, antifungal, antiparasitic, antidiabetic, anticancer, antioxidant activity along with several potential chemical compounds that could be of significant importance in the clinical sector. As per the literature search on various databases such as PubMed, most of the previously published articles are reported on the biological activity of *Terminalia* sp. During the year 2010 to 2020, and this is presented in **Figure 3** (https://pubmed.ncbi.nlm.nih.gov/?term=*Terminalia*+sp.&filter=ds1.y_10, 15 July 2020).

It has been proved that the phytochemicals presented in the plant species have been the most popular research nitch investigated thus far (**Figure 3**). The plant species is worldwide distributed, with more than 250 species in Asia, Himalayas, Madagascar, Australia, and Southern Africa. Importantly, the ethnopharmacological use of the plant species is well-known in India named Ayurvedic medicine as well as in Chinese medicine. Also, some of the *Terminalia* sp. fruits including *Terminalia bellirica* (Gaertn.) Roxb. and *Terminalia chebula* Retz. have been used as a polyherbal formulation in Ayurvedic and Thai folk medicine named Triphala (Intharuksa et al., 2016). Therefore, *Terminalia* sp. phytochemicals have been studied by many researchers. Several phytochemical groups including glycosides, flavonoids, tannins, phenols, saponin, carbohydrates, and proteins have been found in the plant species (Abraham et al., 2014). Furthermore, pure new compounds and pure well-known compounds have also been isolated from several *Terminalia* sp. (Wright et al., 2016). According to the varieties of chemical constituent compounds present in the *Terminalia* plant species, a study on the phytochemicals of the *Terminalia* sp. including the isolation of a new compound to be used as a novel drug for the treatment of diseases is an important area of research. The antibacterial activity of *Terminalia* sp. is the second popular research determined by the researchers (**Figure 3**). *Terminalia* sp. has been used in traditional medicine to treat bacterial infectious diseases including diarrhea, dysentery, pneumonia, and sore throats (Eloff et al., 2008). Therefore, the trend of the bioactivity study on *Terminalia* sp. is an important area of research. It has been reported that the growth of airborne pathogens including MDR *Acinetobacter* sp. and MDR *Pseudomonas aeruginosa* is suppressed when the microorganisms were treated with *Terminalia bellirica* (Gaertn.) Roxb. (Dharmaratne et al., 2018) and *Terminalia chebula* Retz. (Sharma et al., 2012) fruit extracts.

Malaria, the number one killer parasitic disease, is the world’s most significant protozoan disease. It has been reported by the World Health Organization that 228 million cases of malaria worldwide occurred in 2018. In addition, *Haemonchus contortus* and *Trypanosoma brucei* are some of the major causes of human morbidity and mortality in Africa and Some part of Asia. It has been noticed that *Terminalia* sp. are one of the most important plant ingredient in traditional medicine to treat several infectious diseases such as malaria (Malterud, 2017). Of this, antiparasitic particularly antimalarial activities of *Terminalia* sp. have been studied across the world. Also, the anticancer activity of *Terminalia* sp. has been focused mainly since cancer is one of the most important human diseases that causes public health concerns worldwide. The diseases are abnormal cell growth with the potential to spread to other organs of the human body. Importantly, cancer is considered one of the major non-communicable diseases leading to high morbidity and mortality rates as well as a huge impact of economical loss on a large scale. In line with this report, the anticancer activity of *Terminalia* sp. has then been a hotspot in search of novel anticancer therapy. Basing on the above insight into the importance of the *Terminalia* sp., it is evident to compile a detailed report on the medicinal potential of the various species of *Terminalia* sp. and its important phytoconstituents and pharmacological importance.

## *Terminalia* sp. Medicinal Potential (Folk Medicinal Uses, Traditional Uses)

The genus *Terminalia* sp. are widely used in various traditional medicines such as traditional Chinese medicine, Tibetan medicine, and Indian Ayurvedic medicine practices (Zhang et al., 2019). *Terminalia* sp. is found to possess various bioactivities such as antitumor, antiinflammatory, anti-bacterial, antifungal, and antiviral properties (Zhang et al., 2019). There are several species of plants belonging to the genus *Terminalia*, and some of these plant species and their traditional uses are discussed below.

*Terminalia argentea* Mart. is an aboriginal tree growing in various regions of Brazil. Leaf of *T. argentea* Mart. is traditionally used to treat digestion and respiratory-related diseases in Brazil. It has also been reported that the hydroethanolic extract from the leaves of *T. argentea* Mart. has no cytotoxicity in CHO-K1 and AGS cells *in vitro* (Beserra et al., 2018). *T. arjuna* (Roxb. ex DC.) Wight & Arn. is propagated by seeds and grows almost in all types of soils, however, humid, fertile loam and red lateritic soils are preferred (Dwivedi and Chopra, 2014). *T. arjuna* (Roxb. ex DC.) Wight & Arn. is an endemic tree widely found in India and Sri Lanka and used traditionally for cardioprotective and hepatoprotective purposes. In the indigenous medicine system, *T. arjuna* (Roxb. ex DC.) Wight & Arn. is widely documented for its use in treating cardiovascular diseases (Kapoor et al., 2014). (2) Arjunolic acid, an oleanane triterpenoid found in the heartwood of *T. arjuna* (Roxb. ex DC.) Wight & Arn., has been demonstrated to contribute to the bioactivities of the plant (Toppo et al., 2018). Also, evidence suggested that *T. arjuna* (Roxb. ex DC.) Wight & Arn., bark administration relieved trinitrobenzenesulfonic acid-induced colitis in an animal model by reducing the expression of pro-inflammatory cytokines and chemokine and decreasing oxidative stress (Cota et al., 2019). Alcoholic extract from the bark of *T. arjuna* (Roxb. ex DC.) Wight & Arn., has been demonstrated to protect against picrotoxin in mice by regulating related genes (Chandra Sekhar et al., 2017). The fruit of *Terminalia bellirica* (Gaertn.) Roxb. is widely documented for its use in treating diseases such as diarrhea, cough, and scorpion-sting, etc. In India, *T. bellerica* (Gaertn.) Roxb. is used to treat diarrhea based on its antioxidant and antibacterial properties (Pandey et al., 2017). It has been found that extracts of *T. bellirica* (Gaertn.) Roxb. fruits possess antibacterial activity without having cytotoxicity (Dharmaratne et al., 2018). A recent study showed that the aqueous acetone extract of *T. bellirica* (Gaertn.) Roxb. fruits attenuate CCl4-induced oxidative stress and liver damage in a rat model (Kuriakose et al., 2017).

*Terminalia brownii* Fresen. has been used to treat brown-erythematous excoriated papules, plaques, and lichenification in the formulation of scented smoke baths (Kibar Ozturk et al., 2018). In traditional medicine in Southeast Asia, the aqueous extract of *Terminalia catappa* L. leaves are used to treat antipyretic, hemostatic, hepatitis, and liver-related diseases *T. catappa* L. is also used to manage diabetic due to its property of reducing oxidative stress, inflammation, angiogenesis, lipid profile correction, and direct hypoglycemic actions (Behl and Kotwani, 2017). Extracts from the leaves of *T. catappa* L. has been reported to attenuate the growth of *Staphylococcus aureus* (ATCC 25923) and *Pseudomonas aeruginosa* (ATCC 27853) (Allyn et al., 2018). Besides, methanolic extracts from *T. catappa* L. are found to prevent hydrogen peroxide-induced oxidative damage in human fibroblasts (Hs68), thereby can be potentially used to manage skin aging (Huang et al., 2018).

*Terminalia chebula* Retz. is widely used to treat dementia, constipation, and diabetes in traditional Indian and Iranian medicine (Jokar et al., 2016). Studies showed that *T. chebula* Retz. has various biological activities, including antimicrobial, antiinflammatory, antioxidant, and antitumor (Zhang et al., 2016). It has been demonstrated that *T. chebula* Retz. fruits are rich in phenolic compounds such as (3) gallic acid, (4) ellagic acid, and (5) corilagin, which hold potent antioxidant, anti-inflammatory, cardiotonic, antibacterial, and anticarcinogenic activities (Fan et al., 2015). A randomized placebo-controlled clinical trial indicated that dietary supplementation with a standardized extract of *T. chebula* Retz. fruit (AyuFlex^®^) relieves the discomfort in osteoarthritis (Lopez et al., 2017). Also, *T. chebula* Retz. is a potent cognitive enhancer for amnesia due to its antioxidant activity (Kim et al., 2018). However, the safety assessment regarding the use of *T. chebula* Retz. in amnesia is lacking (Suganthy et al., 2018). Active components such as (4) ellagic acid are reported to play essential roles in the neuroprotective effect of *T. chebula* Retz. *in vivo* (Shen et al., 2017). *T. chebula* Retz. reduces oxidative cell death induced by PC12 and OLN-93 caused by quinolinate, which suggests the neuroprotection and oligoprotection effects of *T. chebula* Retz. (Sadeghnia et al., 2017). Another study suggested that *T. chebula* Retz. extract attenuates inflammation in microglial cells; therefore, it can be used as a potential anti-inflammatory agent for the treatment of inflammatory diseases of the central nervous system (Rahimi et al., 2018). In addition, the antioxidant activity of *T. chebula* Retz. has been demonstrated in *in vitro* models in previous studies (Kumar et al., 2018).

It has been reported that the methanolic extract of *Terminalia coriacea* (Roxb.) Wight & Arn. (*Terminalia coriacea* Spreng.) leaves reduce the paw edema and the weights of granulomatous tissue in both acute and chronic *in vivo* inflammatory models (Khan et al., 2018). *Terminalia cunninghamii* C.A.Gardner, is a native nut traditionally used by Australian Indigenous peoples for oxidant-related issues (Zhong et al., 2018). The fruit and leaf extracts of *Terminalia ferdinandiana* Exell, an endemic Australian plant, have been found to possess strong antibacterial activity against various bacterial pathogens (Cheesman et al., 2019). The leaf extract of *T. ferdinandiana* Exell has a potent growth inhibition effect on plantar malodor-producing bacteria (Mcmanus et al., 2017). It has also been reported that extracts from *T. ferdinandiana* Exell down-regulate the growth of *Shewanella* sp., which are essential causes of fish spoilage (Wright et al., 2019). *Terminalia laxiflora* Engl. contains a wide variety of antimycobacterial compounds, including ellagitannins, ellagic acid derivatives, triterpenes, fatty acids, and fatty alcohols (Salih et al., 2018). Evidence indicates that the fungal extract from *T. laxiflora* Engl. affects the NF-κB signaling pathway in K562 myelogenous leukemia cell line (Tawfike et al., 2018). In Mali, *Terminalia macroptera* Guill. & Perr. is one of the most widely used plants for malaria in traditional medicine (Pham et al., 2014). The first *in vitro* *T. macroptera* Guill. & Perr. study in 1996 found that the roots and leaves of *T. macroptera* Guill. & Perr. hold antibacterial activity (Silva et al., 1996). The safety of the use of *T. macroptera* Guill. & Perr. in malaria was further confirmed in the following studies using an *in vivo* Albino Swiss mice model (Haidara et al., 2018). *Terminalia sericea* Burch. ex DC. is traditionally used in the treatment of stomach ailments, infections, hypertension, and diabetes mellitus (Busisani et al., 2018). Recent studies in medical sciences have revealed that the potential of *T. sericea* Burch. ex DC. includes: antiviral, antibacterial, antitumor, antiinflammatory, antioxidant, and wound healing activities (Fan et al., 2015).

## Phytochemistry and Chemical Constituents of *Terminalia* sp.

### Phytochemistry

*Terminalia sericea* Burch. ex DC. is a medicinal plant used mainly to treat diarrhea, sexually transmitted infections, skin rashes, tuberculosis, and other infections. Its biological activities are due to valuable phytochemicals, including triterpenes, alkaloids, and flavonoids (Mongalo et al., 2016). Tannins and polyphenols are two major active components of the plant, which contribute to the bioactivity of *Terminalia* sp. (Li et al., 2011). Tannins are a kind of polyphenolic compounds that can be classified into three groups based on their structures: hydrolyzable, condensed, and complex tannins (Chang et al., 2019). It is reported that other ingredients, including triterpenoids, flavonoids, and aliphatic compounds, have bioactive properties (Chang et al., 2019). *Terminalia catappa* L. that has antiHIV features was analyzed for phytochemicals by direct binding assay with mass spectrometry (MS) techniques (Dwevedi et al., 2016). These authors described the presence of tannins, gallotannins, ellagitannins, cyanidin, and flavonoids. More recently, chemical analysis of *Terminalia catappa L.* bark, and leaves performed by Tercas et al. (Terças et al., 2017), reported hydrolyzable tannins (6) punicallin, (7) punicalagin, (3) gallic acid, and flavonoid C-glycosides. These authors have used techniques like gas chromatography coupled to mass spectrometry with electron impact (GC/MS/EI), high-performance liquid chromatography coupled to mass spectrometry “electrospray” ionization in positive mode (HPLC/MS/MS/ESI+) and hydrogen nuclear magnetic resonance (1HNMR). In addition, leaf extract fractions showed antifungal properties against *Candida* sp.

Recently, Wright and the team explored the antimicrobial properties of *Terminalia ferdinandiana* Exell (Kakadu plum) extracts against *Shewanella* sp. growth, the main cause of fish spoilage (Wright et al., 2019). Several compounds were identified by LC-MS analysis that displayed this pharmacological application. The aqueous extract of *Terminalia bellirica* (Gaertn.) Roxb. from fruits have been reported to contain glycosides, flavonoids, tannins, phenols, saponin, carbohydrates, and proteins (Abraham et al., 2014). Proteins, carbohydrates, and tannins were found in both aqueous, and methanol extracts of *Terminalia chebula* Retz. leaves. Alkaloids were present in both aqueous and methanol extracts of the fruit, while flavonoids were detected in both aqueous and methanol extracts of all parts of *Terminalia chebula* Retz. except root (Vemuri et al., 2019). (8)Terflavin B and (9) chebulinic acid were pure compounds isolated from *Terminalia chebula* Retz. fruits (Cock, 2015). *Terminalia schimperiana* Hochst. ex Engl. & Diels (synonym of *Terminalia glaucescens* Planch. ex Benth.) root bark was reported to contain flavonoids, tannins, steroids, carbohydrates, and terpenoids in n-hexane, ethylacetate, and methanol as solvents (Khan et al., 2019). Besides, (10)stigmasterol was a pure compound isolated from the root bark of *Terminalia schimperiana* Hochst. ex Engl. & Diels (synonym of *Terminalia glaucescens* Planch. ex Benth.). The pure compound was a white-yellow crystal, characterized by spectral techniques such as 1H-NMR, 13C-NMR, COSY, HSQC, and HMBC spectral techniques (Khan et al., 2019). Aqueous and methanol extracts of *Terminalia grandiflora* Benth. nut, fruit, and leaf contained high levels of water-soluble phenolics, moderate to high levels of tannins, and low levels of flavonoids and anthraquinones; while *Terminalia carpentariae* C.T.White (synonym of *Terminalia hadleyana* subsp. *carpentariae* (C.T.White) Pedley), leaf extract showed high levels of water-soluble phenolics and tannins (Wright et al., 2016). Furthermore, the methanol extract of *Terminalia grandiflora* Benth. nut and *Terminalia carpentariae* C.T.White [synonym of *Terminalia hadleyana* subsp. *carpentariae* (C.T.White) Pedley], leaf exhibited antibacterial activity against *Bacillus anthracis* with MIC at 155 and 74 µg/mL, respectively (Wright et al., 2016). Pure compounds including (11) methyl N-hydroxybenzenecarboximidoate; (12) 1-octen-3-ol; (13) 5-hepten-2-one; (14) 6-methyl-, 2-tert-butoxyethanol; (15) 2-ethyl-1-hexanol; (16) dimethyl succinate; (17) isophorone; (18) α-citronellol; (19) nonanal; (20) 4-oxoisophorone; (21) ethyl benzoate; (22) methyl benzeneacetate; (23) α-terpineol; (24) 2-isopropylidene-3-methylhexa-3,5-dienal; (25) lauraldehyde; (26) 2,4-dimethyl-benzaldehyde; (27) 1,3-pentanediol; (28) 2,2,4-trimethyl-, 1-isobutyrate; (29) 2,4-di-tert-butylphenol; (30) ethyl para-ethoxybenzoate; and (31) 2,2,4-trimethyl-1,3-pentanediol diisobutyrate were detected from the methanol *Terminalia carpentariae* C.T.White (synonym of *Terminalia hadleyana* subsp. *carpentariae* (C.T.White) Pedley), leaf extract using GC-MS headspace analysis (Wright et al., 2016). It has been highlighted that (32) 2-(1,1-dimethylethoxy)-ethanol; (33) caryophyllene; (31) 2,2,4-trimethyl-1,3-pentanediol diisobutyrate; and (34) butyl octyl phthalate were detected in *Terminalia grandiflora* Benth. methanol extract but they were not found in the *Terminalia carpentariae* C.T.White (synonym of *Terminalia hadleyana* subsp. *carpentariae* (C.T.White) Pedley), leaf extract (Wright et al., 2016). Groups of flavonoids, including (35) quercetin, (36) kaempferol, tannins, saponins, and phytosterols presented in the *Terminalia catappa* L. fruit, have been documented (Cock, 2015). This information indicates varieties of chemical compounds present in *Terminalia* plant species. This study shows that there are several chemical compounds in *Terminalia* plant species. As such, assessment and isolation of phytochemicals from *Terminalia* sp. could hold promise in the discovery of novel new compounds leading to the development of new drugs for the management and control of diseases.

### Chemical Constituents of *Terminalia* sp.

The stem bark of *T. arjuna* (Roxb. ex DC.) Wight & Arn. contains high levels of antioxidant compounds, including glycosides, flavonoids, tannins, and inorganic minerals. Amongst the terpenoids reported in *T. arjuna* (Roxb. ex DC.) Wight & Arn. bark is (37) β-sitosterol, (38) terminic acid (**Table 1**) (Anjaneyulu and Prasad, 1982), (39) terminoside A (Ali et al., 2003a; Ali et al., 2003b), and (40) arjunaphthanoloside (**Table 1**). (39) Terminoside A and (40) arjunaphthanoloside are particularly interesting due to their reported therapeutic activities. Terminoside A inhibits the production of nitric oxide and decreases the levels of nitric oxide synthase in macrophages stimulated by lipopolysaccharides, and arjunaphthanoloside has strong antioxidant activity (Ali et al., 2003a; Ali et al., 2003b). *T. arjuna* (Roxb. ex DC.) Wight & Arn. bark also contains very high levels of antioxidant flavonoid compounds compared to other plants (Nair et al., 1996; Nair and Nagar, 1997). These flavonoids include (41) arjunolone, (42) bicalein, flavones, (36) kampferol, (43) pelorgonidin, (35) quercetin, (Sharma et al., 1982), and (48) luteolin, (Pettit et al., 1996). There is evidence that similar bioflavonoids prevent oxidation of LDL cholesterol through its free radical scavenging activity (Fuhrman and Aviram, 2001), inhibit endothelial activation (Carluccio et al., 2003) and inhibit platelet aggregation (Ruf, 1999). They also have cyclooxygenase inhibitory activity and, therefore, can prevent thrombosis (Ruf, 1999). Kuo et al. (2005), reported (44) castalagin, (8) terflavin, and (45) terchebulin while Lin et al. (2000), reported (44) castalagin from *T. arjuna* (Roxb. ex DC.) Wight & Arn.

**Table 1:** Chemical Constituents of *T. arjuna* (Roxb. ex DC.) Wight & Arn.

Compound No.	Chemical Constituents name	Structure	Reference
(37)	β-sitosterol	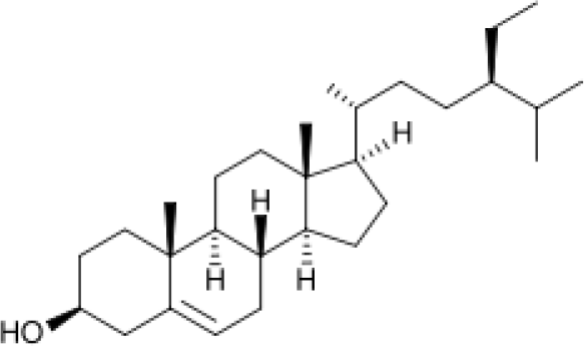	(Anjaneyulu and Prasad, 1982)
(38)	Terminic acid	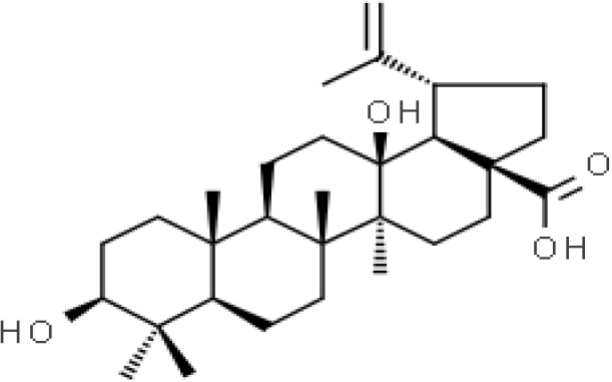	(Anjaneyulu and Prasad, 1982)
(48)	Luteolin	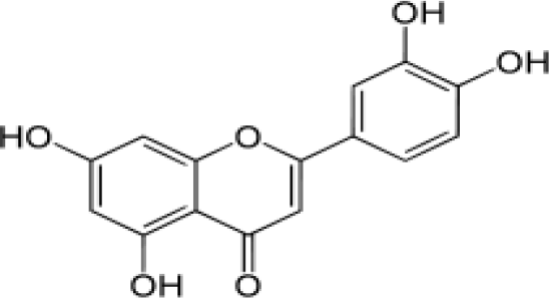	(Pettit et al., 1996)
(44)	Castalagin	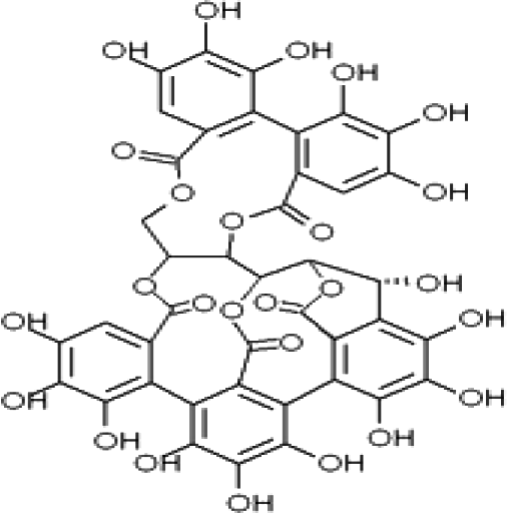	(Kuo et al., 2005)
(8)	Terflavin	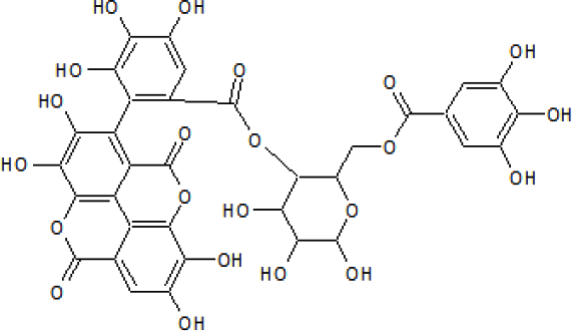	(Kuo et al., 2005)
(45)	Terchebulin	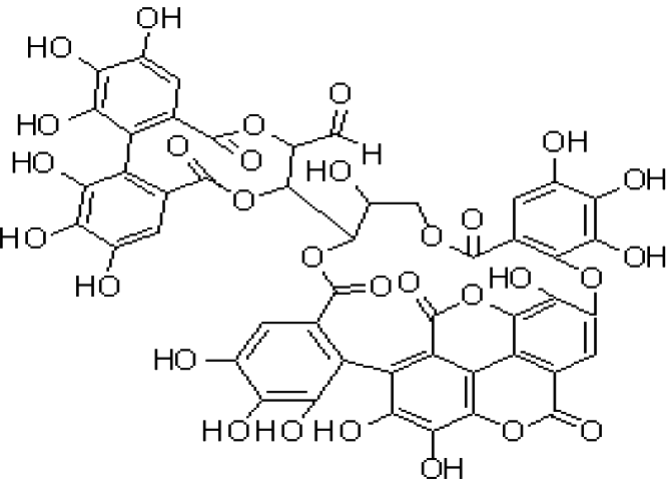	(Kuo et al., 2005)
(6)	Punicallin	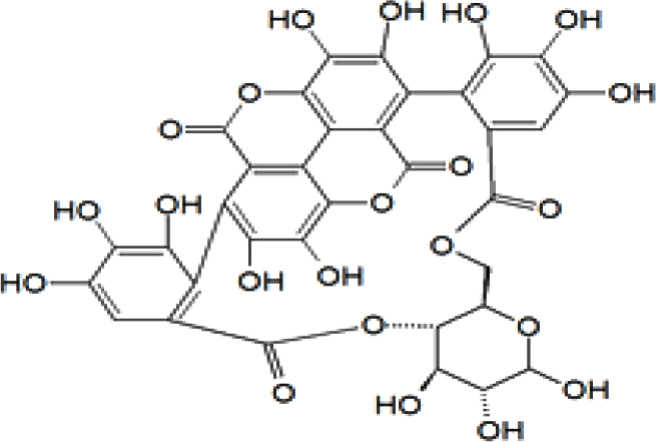	(Lin et al., 2000)
(43)	Pelorgonidin	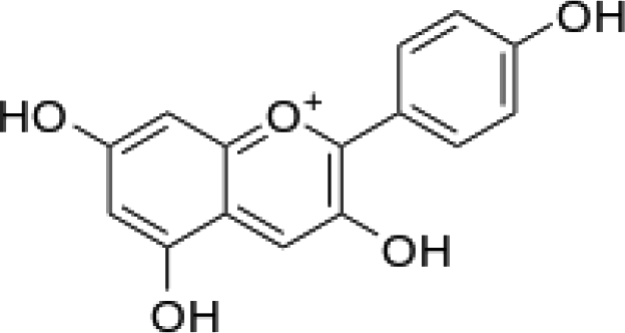	(Sharma et al., 1982)
(42)	Bicalein	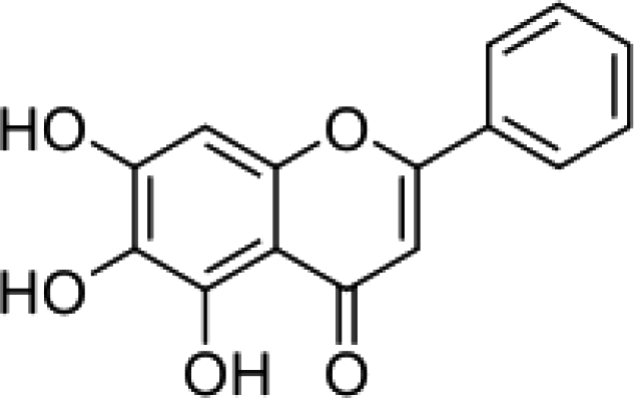	(Sharma et al., 1982)
(41)	Arjunolone	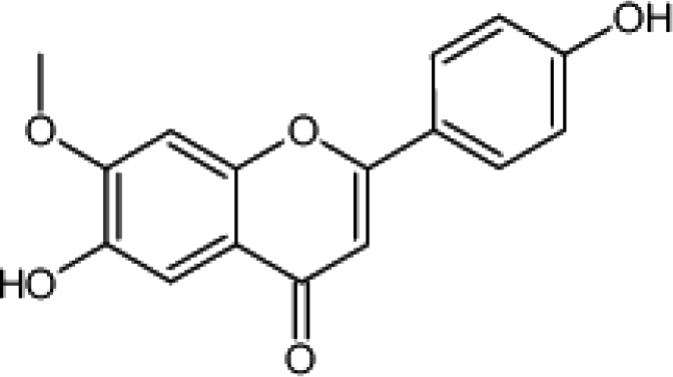	(Sharma et al., 1982)
(39)	Terminoside A	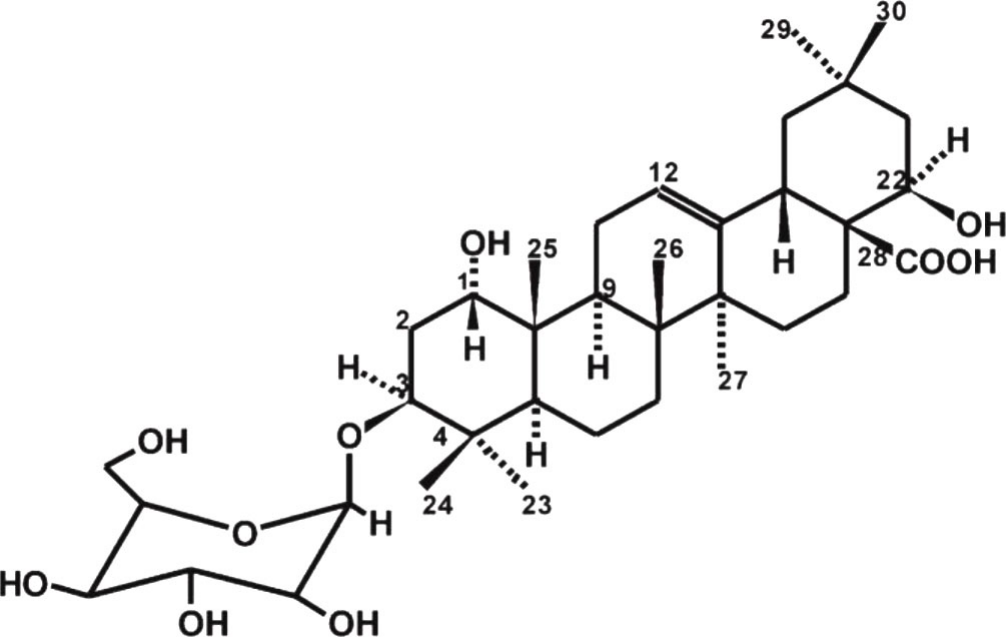	(Ali et al., 2003a; Ali et al., 2003b)

*T. ferdinandiana* Exell fruit is the main source of vitamin C, in the health food, cosmetic, and pharmaceutical industries. However, *T. ferdinandiana* Exell fruit also contains many other compounds that also contribute to its high antioxidant activity (Netzel et al., 2007; Konczak et al., 2010). Although many of these compounds have not been identified yet, *T. ferdinandiana* Exell fruit has been shown to contain benzoic acids, flavanols, or flavanones (Konczak et al., 2010). *T. ferdinandiana* Exell fruit is a good source of (3) gallic acid and (4) ellagic acid (**Table 2**) (Cunningham et al., 2009), which also demonstrates strong antioxidant activity *in vitro* (Ohno et al., 1999; Losso et al., 2004). *T. ferdinandiana* Exell fruit is also very rich in (36) chlorophyll a and (47) chlorophyll b (**Table 2**), which have previously been shown to be capable of quenching oxidative stress. Lipophilic *T. ferdinandiana* Exell fruit extracts are also rich in (48) luteolin and with (49) vitamin E (**Table 2**) and vitamin E analogs (Konczak et al., 2010). (50) Hesperitin (**Table 2**), as well as the glycosides (36) kaempferol, (48) luteolin, and (35) quercetin (**Table 2**), are some of the other antioxidants present in *T. ferdinandiana* Exell fruit (Konczak et al., 2010). *T. ferdinandiana* Exell fruit is also a good source of the minerals magnesium, zinc, calcium, potassium, sodium, iron, phosphorous, manganese, copper, and molybdenum (Konczak et al., 2010).

**Table 2:** Chemical Constituents of *T. ferdinandiana* Exell.

Compound No.	Chemical Constituents name	Structure	Reference
(51)	Ascorbic acid	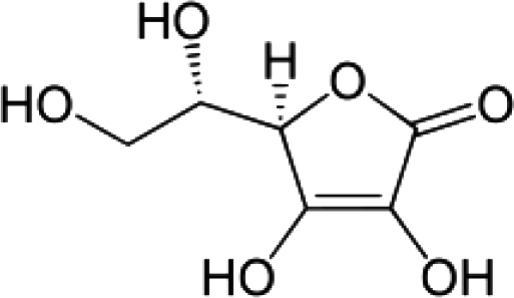	(Netzel et al., 2007; Konczak et al., 2010)
(3)	Gallic acid	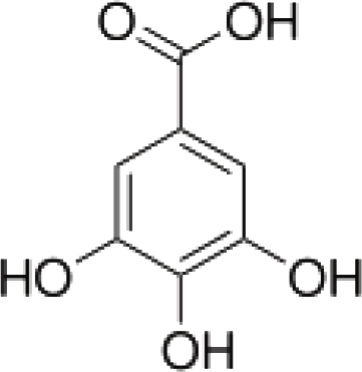	(Cunningham et al., 2009)
(35)	Quercetin	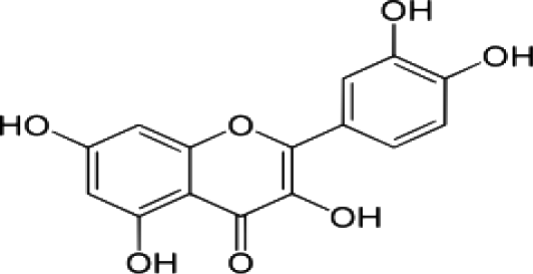	(Konczak et al., 2010)
(52)	α-tocopherol	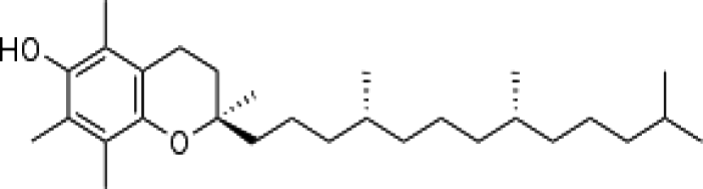	(Konczak et al., 2010)
(4)	Ellagic acid	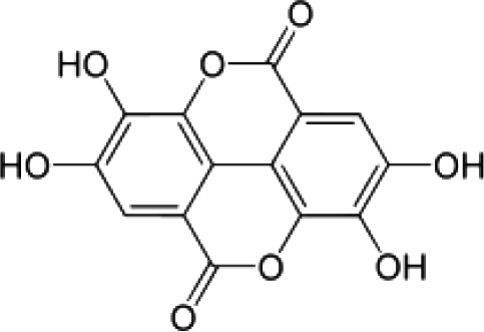	(Cunningham et al., 2009)
(48)	Luteolin	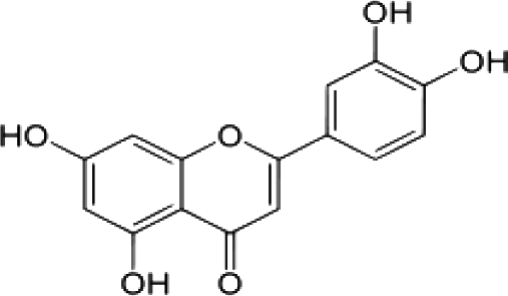	(Konczak et al., 2010)
(36)	Kaempferol	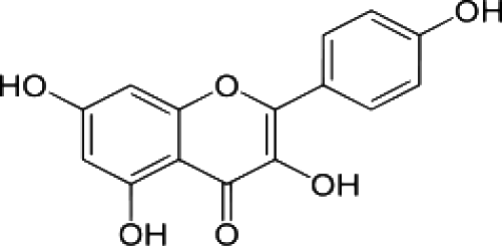	
(50)	Hesperitin	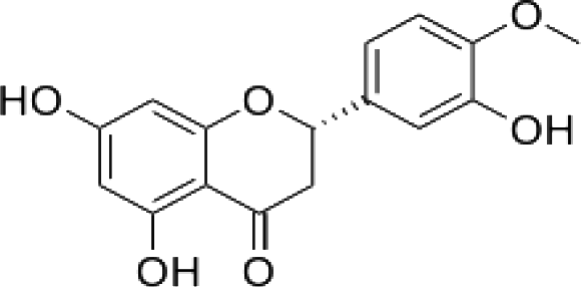	
(46)	Chlorophyll a	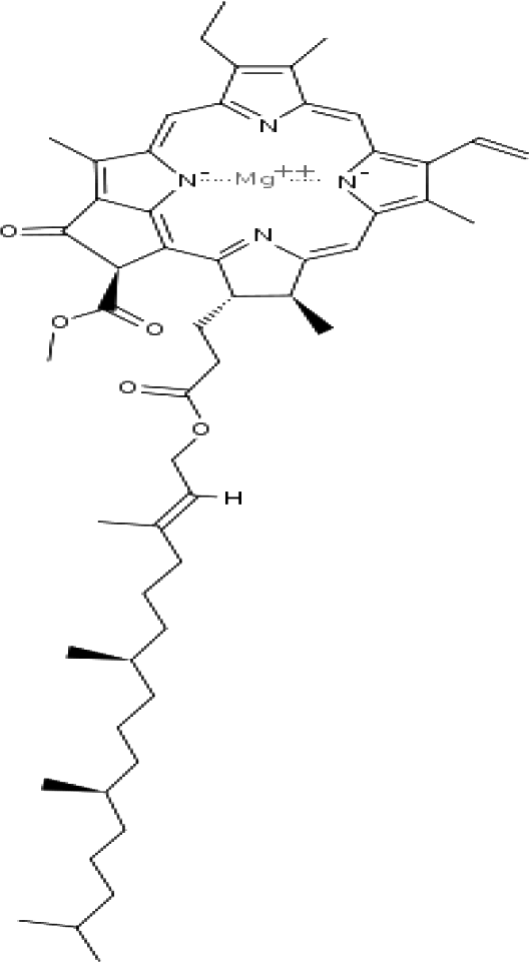	
(47)	Chlorophyll b	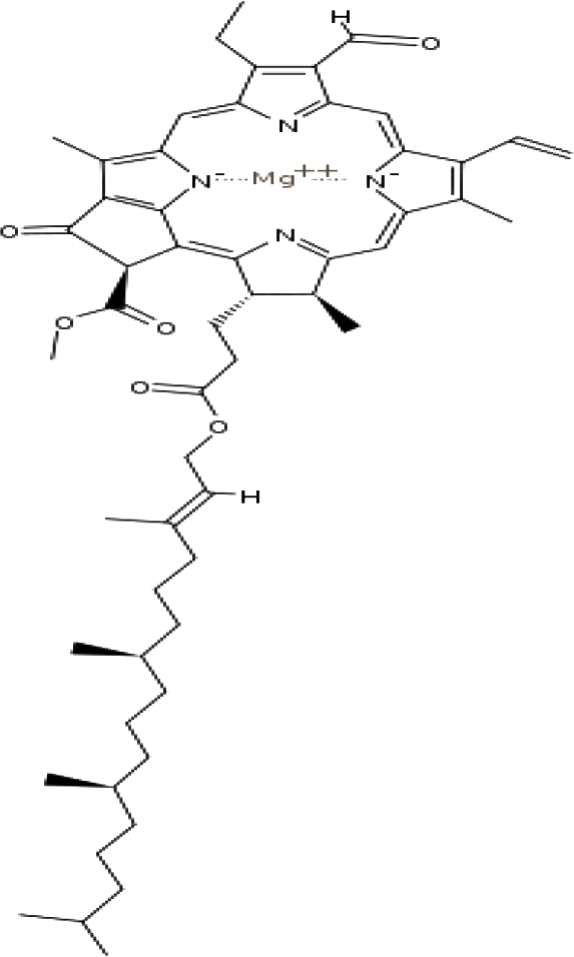	

## Pharmacological Effects of Active Constituents From *Terminalia* sp.

There is a rising global interest in ethno-pharmacological studies on plants that have been traditionally used to alleviate a myriad of diseases (World Health Organization, 1999; World Health Organization, 2018). Due to the increasing number of people with non-communicable diseases, which represent about 70% of deaths in the world, the search for new pharmacological agents started to evaluate the medicinal plants mentioned in the pharmacopeias of different countries (World Health Organization, 1999; World Health Organization, 2018). In this sense, *Terminalia* sp. has been the center of attention of many studies, which aim to evaluate and validate the therapeutic potential based on its ethnobotanical use. As *Terminalia* species can be found in many countries, the ethnobotanical information on their medicinal use is diverse; for example fruits, leaves, and stem bark of different *Terminalia* sp. has been reported to be used as a remedy to treat geriatric diseases, memory improvement, abdominal and back pain, cough, and colds, conjunctivitis, diarrhea, fever, headache, heart disorders, inflammation, leprosy, pneumonia, sexually transmitted diseases, urinary disorders, among others (Maulik and Katiyar, 2010; Maulik and Talwar, 2012; Cock, 2015; Afshari et al., 2016). Medicinal plants have long been known to be rich sources of bioactive compounds. *Terminalia* sp. belongs to the family *Combretaceae*, has several biological activities, including antibacterial, antifungal, as well as antiparasitic. In addition, the extracts from the plant species showed antidiabetic, anticancer, and antioxidant activity.

*Antidiabetic and Antiobesity Studies of Terminalia* sp.

The International Diabetes Federation states that type-2 diabetes is a chronic condition that occurs when there are raised levels of plasma glucose, attributed to insufficient/lack production of insulin, or because the body cannot use the insulin it produces (International Diabetes Federation, 2019). It is estimated that around 463 million people live with diagnosed diabetes, and this number is expected to rise to 700 million by 2045. The first line of treatment of diabetes is a combination of exercise, changes in lifestyle, dietary modification, and drug prescription; sulfonylureas and meglitinides, metformin, PPAR antagonists (like thiazolidinediones), α-glucosidase inhibitors, among others (Defronzo et al., 2015). However, people from low- and middle-income countries have restricted access to pharmaceutical treatment due to economic restraints; thus, they sometimes use alone or in combination different medicinal plants to treat hyperglycemia, diabetes and some of its complications (World Health Organization, 1999; Mata et al., 2013). Studies regarding the potential antidiabetic properties of *Terminalia* sp. have been consistent from the last 10 years (according to the Web of Science and Scopus databases); however, it is important to mention that most of these reports represent only *in vitro* studies. This section aims to gather recent studies with *Terminalia* sp. and their potential antidiabetic activity.

A study by Yang et al. (2013), evaluated fruits from two different *Terminalia* sp., *T. chebula* Retz., and *T. bellerica* (Gaertn.) Roxb., and an Ayurvedic formulation from the two species. The authors showed that the compounds (53) chebulagic acid; (5) corilagin; (54) 2,3,6-tri-O-galloyl-β-glucose; (55) 1,2,3,6-tetra-O-galloyl- β-D-glucose found in *T. bellerica* (Gaertn.) Roxb. and the formulation enhanced the signaling of 8Peroxisome proliferator-activated receptors (PPARα and PPARγ). A structure-dependent effect was shown when only the gallotannins, (56) 1,2,3,6-tetra-O-galloyl- β-D-glucose and (57) 1,2,3,4,5-penta-O-galloyl- β-D-glucose enhanced up to 9.92-fold the cellular glucose uptake in HepG2 cells and inhibited the rosiglitazone-induced adipogenesis (Yang et al., 2013). A similar report by Shyni et al. (2014), showed the potential of (53) chebulagic acid to enhance glucose transport in adipocytes using 3T3-L1 preadipocytes to elucidate its PPARγ agonistic effect. To achieve this, the authors used (53) chebulagic acid from *Terminalia chebula* Retz. using 3T3-L1 cells at concentrations of 10, 50, and 100 µM. The importance of research for PPARγ antagonists from plant origin is to elucidate active molecules that partially inhibit these molecules, without the adverse side effects caused by conventional PPARγ antagonists (Shyni et al., 2014). The authors concluded that (53) chebulagic acid from *T. chebula* Retz. enhanced the expression of C/EBPα, a PPARγ target in adipocytes, but not at the extent of rosiglitazone.

Mopuri et al. (2015), evaluated a different *Terminalia* species, *T. paniculata* Roth from India. The study tested the antiobesity effect of ethanolic extracts from barks of *T. paniculata* Roth on male Sprague-Dawley rats. Of the evaluated parameters, *Terminalia* extracts reduced body weight, lean mass, total fat, fat percentage, decreased glucose blood levels at 60 min, reversed insulin resistance, and lowered serum cholesterol, triglycerides, and low-density lipoproteins. The authors concluded that the potential antiobesity effect of barks from *T. paniculata* Roth might be attributed to the down-regulation of the expression of lipogenic genes and leptin; also, and up-regulation of adiponectin and AMPK-1α. The potential hypolipemic effect of *Terminalia chebula* Retz. has been assessed by Reddy et al. (2015). The authors used methanolic bark extracts of *T. chebula* Retz. at concentrations of 5, 50, 300, and 2000 mg/kg. The toxicity of these extracts was evaluated using 8–12 weeks old female rats at the doses mentioned above. Reddy et al. (2015), report no observed toxicity effect with any of the tested concentrations. Furthermore, the administration of *T. chebula* Retz. bark extract at 600 mg/kg significantly caused a hypolipidemic effect in high-cholesterol hyperlipidemic rats, which was evidenced by increased serum high-density lipoprotein cholesterol levels.

Another mechanism in which *Terminalia* sp. has shown its antiobesity potential is through the inhibition of glucose metabolic enzymes such as α-glucosidase. For instance, Pham et al. (2014), evaluated *Terminalia macroptera* Guill. & Perr. extracts glucosidase inhibitory capacity. Chromatographic analysis showed the presence of the polyphenols (53) chebulagic acid, (73) chebulic acid trimethyl ester, (71) corilagin, (70) methyl gallate, (74) narcissin, and (118) rutin. Methanol, ethyl acetate, and butanol *Terminalia* sp. extracts were outstanding inhibitors of α-glucosidase with IC_50_ values of 0.47, 0.4, and 0.4 µM, respectively. Moreover, (53) chebulagic acid isolated from extracts showed an inhibitory rate with IC_50_ values of 0.05 µM. Similarly, Nguyen et al. (2016), studied the enzymatic inhibitory potential of extracts of trunk-bark of three *Terminalia* species, *T. alata* Roth*, T. bellerica* (Gaertn.) Roxb. and *T. corticosa* Pierre ex Laness.

*Terminalia* species showed inhibitory activity against α-amylase and α-glucosidase, and lowered fasting blood glucose in streptozotocin-induced diabetic rats. *T. bellerica* (Gaertn.) Roxb. extracts showed the most potent inhibitory activity against α-glucosidase, followed by *T. corticosa* Pierre ex Laness, and *T. alata* Roth, with IC_50_ values of 0.41, 1.42, and ≥ 4 mg/mL, respectively. The authors found a positive correlation between the polyphenolic content of *Terminalia* species (as evaluated by spectrophotometry) and the α-glucosidase inhibitory capacity of the extracts. Moreover, Makihara et al. (2012), evaluated hot water extracts of fruits from *Terminalia bellirica* (Gaertn.) Roxb. on obesity-related disorders. The authors suggest that *Terminalia* sp. has a preventive effect on obesity, insulin resistance, and hyperlipidemia in spontaneously obese type 2 diabetic mice. *Terminalia* sp. treatment does not affect food intake; however, it slightly suppressed body weight gain (Makihara et al., 2012). Moreover, *T. bellerica* (Gaertn.) Roxb. treatment significantly suppressed in a dose a time-dependent manner, the accumulation of visceral and subcutaneous fat after the 7th week. *Terminalia* treatment also improved plasma and hepatic lipid levels, as it was observed by the decreased LDL/HDL ratio from 0.28 to 0.23 in *Terminalia* sp. at 3% treated mice. The authors managed to identified that gallic acid might be the responsible active compound for the inhibition of lipid absorption, as it is a potent pancreatic lipase inhibitor. As a follow-up of the study, Makihara et al. (2016), studied the anti-obesity mechanism of gallic acid of fruits from *Terminalia bellirica* (Gaertn.) Roxb. by evaluating adipocyte3 differentiation using mouse 3T3-L1 cells. Gallic acid at concentrations of 10–30 µM enhanced the expression and secretion of adiponectin *via* adipocyte differentiation, which is enhanced by *T. bellerica* (Gaertn.) Roxb. extracts. Gallic acid enhanced the expression of the mRNA encoding for the marker of adipocyte differentiation Fabp4.

**Figure 4:**
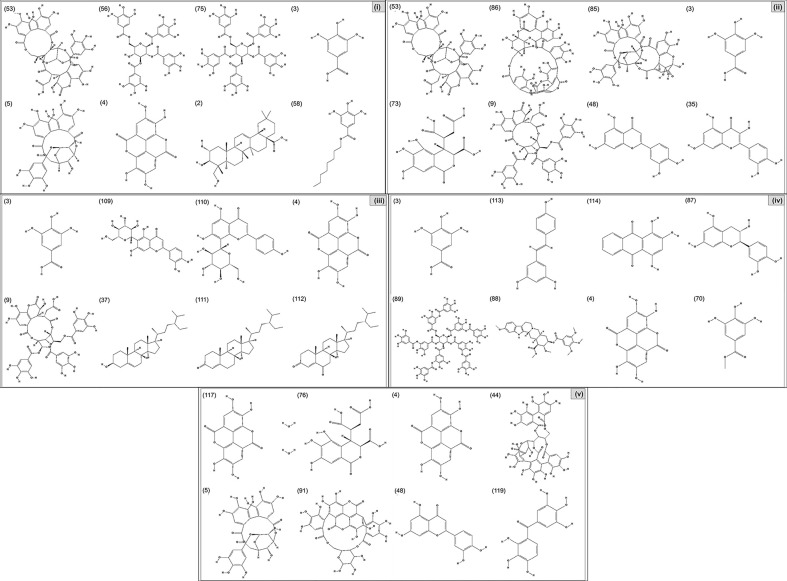
(i) Compounds from *Terminalia* species that have been studied for their antidiabetic and antiobesity properties. (53) Chebulagic acid, (56) 1,2,3,6-tetra-O-galloyl-β-D-glucose, (75) 1,2,3,4,6-penta-O-galloyl-beta-D-glucopyranose, (3) gallic acid, (5) corilagin, (4) ellagic acid, (2) arjunolic acid, (58) octyl gallate. Images from the National Center for Biotechnology Information, 2019a; National Center for Biotechnology Information, 2019c; National Center for Biotechnology Information, 2019g; National Center for Biotechnology Information, 2019i; National Center for Biotechnology Information, 2019k; National Center for Biotechnology Information, 2019m; National Center for Biotechnology Information, 2019r; National Center for Biotechnology Information, 2019s; (ii) Compounds from *Terminalia* species that have been studied for their antiproliferative properties. (53) Chebulagic acid, (86) punicalagin, (85) geraniin, (3) gallic acid, (73) chebulic acid, (9) chebulinic acid, (48) luteolin, (35) quercetin. Images from the National Center for Biotechnology Information, 2019c; National Center for Biotechnology Information, 2019d; National Center for Biotechnology Information, 2019f; National Center for Biotechnology Information, 2019h; National Center for Biotechnology Information, 2019m; National Center for Biotechnology Information, 2019n; National Center for Biotechnology Information, 2019p; National Center for Biotechnology Information, 2019v; (iii) Compounds from *Terminalia* species that have been studied for their antiinflammatory properties. (3) Gallic acid, (109) luteolin-6-C-glucoside, (110) vitexin, (4) ellagic acid, (9) chebulinic acid, (37) β-sitosterol, (111) β-sitostenone, and (112) stigmast-4-ene-3,6-dione. Images adapted from National Center for Biotechnology Information, 2019aa; National Center for Biotechnology Information, 2019ad; National Center for Biotechnology Information, 2019b; National Center for Biotechnology Information, 2019f; National Center for Biotechnology Information, 2019k; National Center for Biotechnology Information, 2019m; National Center for Biotechnology Information, 2019o; National Center for Biotechnology Information, 2019z; (iv) Compounds from *Terminalia* species that have been studied for their antioxidant properties. (3) gallic acid, (113) resveratrol, (114) purpurin, (87) catechin, (89) tannic acid, (88) reserpine, (4) ellagic acid, (70) methyl gallate. Images adapted from National Center for Biotechnology Information, 2019ab; National Center for Biotechnology Information, 2019e; National Center for Biotechnology Information, 2019k; National Center for Biotechnology Information, 2019m; National Center for Biotechnology Information, 2019q; National Center for Biotechnology Information, 2019u; National Center for Biotechnology Information, 2019w; National Center for Biotechnology Information, 2019x; National Center for Biotechnology Information, 2019y; (v) Compounds from *Terminalia* species that have been studied for their antimicrobial properties. (117) ellagic acid dihydrate, (76) chebulic acid, (4) ellagic acid, (44) castalagin, (5) corilagin, (91) punicalin, (48) luteolin, (119) exifone. Images adapted from National Center for Biotechnology Information, 2019ac; National Center for Biotechnology Information, 2019d; National Center for Biotechnology Information, 2019i; National Center for Biotechnology Information, 2019j; National Center for Biotechnology Information, 2019k; National Center for Biotechnology Information, 2019l; National Center for Biotechnology Information, 2019p; National Center for Biotechnology Information, 2019t.

Bioassay-guided fractionation of *Terminalia bellirica* (Gaertn.) Roxb. fruit extracts by Latha and Daisy (2013), isolated and identified (58) octyl gallate by 1H and 13C NMR, IR, and mass spectrometry. Also, they reported it as the active compound in *T. bellerica* (Gaertn.) Roxb. responsible for its bioactive potential when examined for its antidiabetic potential. An *in vivo* assay showed that (58) octyl gallate at concentrations of 5, 10, and 20 mg/kg significantly reduced plasma glucose in a dose-dependent manner in diabetic mice. However, the plasma glucose of normal rats treated with the highest (58) octyl gallate concentration was not altered, indicating a normoglycemic effect. This may be attributed to improved proinsulin processing and potentiation of insulin secretion and release from β-cells. This effect is hypothesized as (58) octyl gallate has been reported as a calcium channel and cAMP modulator (Latha and Daisy, 2013). The summary of different *Terminalia* species with antiobesity and antidiabetic potential is given in **Table 3**. In conclusion, phytochemicals such as phenolic compounds, polyphenols, and terpenoids are among the active compounds in *Terminalia* species with antidiabetic and antiobesity potential [**Figure 4(i)**].

**Table 3:** Summarization of the antiobesity and antidiabetic studies of different *Terminalia* species.

*Terminalia* sp.	Mechanism/Mode of action	Phytochemicals (compound no.)	Reference
*T. bellerica* (Gaertn.) Roxb.	Enhancement of PPARα and PPARγ, increased insulin-stimulated glucose uptake. 1,2,3,6-tetra-O-galloyl- β-D-glucose showed the most potent increased in cellular glucose uptake	(53) Chebulagic acid; (55) 1,2,3,6-tetra-O-galloyl-β-D-glucose; (57) 1,2,3,4,5-penta-O-galloyl- β-D-glucose; (59) daucosterol, (3) gallic acid	(Yang et al., 2013)
*T. chebula* Retz.		(59) Arjunetin; (60) arjungenin; (61) arjunglucosides; (62) (63) chebuloside II; (64) shikimic acid	
*T. bellerica* (Gaertn.) Roxb. and *T. chebula* Retz.		(5) Corilagin; (54) 2,3,6-tri-O-galloyl-β-glucose	
*T. chebula* Retz.	mRNA expression of C/EBP-α, a target gene for PPARy, increased with chebulagic acid treatment	(53) Chebulagic acid	(Shyni et al., 2014)
*T. paniculata* Roth	Reduced expression of lipogenic genes (FAS, SREBP-1c, PAPRy, and leptin), up-regulation of adiponectin and AMPK-1α	(4) Ellagic acid; (65) 2’,5’,5,7-tetramethoxy-8-methylflavanone; (66) 3,3’-di-O-methyl ellagic acid; (2) arjunolic acid; (67) galloylarjunolic acid; (68) termilignan; (69) betulinic acid	(Mopuri et al., 2015)
*T. chebula* Retz.	Increased levels of serum high-density lipoprotein cholesterol levels in hypercholesterolemic rats	Not identified	(Reddy et al., 2015)
*T. macroptera* Guill. & Perr.	The inhibitory capacity of α-glucosidase	(64) Shikimic acid, (70) methyl gallate, (71) coriliagin, (53) chebulagic acid, (72) chebulagic acid trimethyl ester, (118) rutin, (74) narcissin	(Pham et al., 2014)
*T. alata* Roth	The inhibitory capacity of α-glucosidase (IC_50_ ≥ 4 mg/mL)	Not identified	(Nguyen et al., 2016)
*T.belirica* (Gaertn.) Roxb.	Inhibitory capacity of α-glucosidase (IC_50_ ≥ 0.41 mg/mL)	Not identified	
*T. corticosa* Pierre ex Laness	The inhibitory capacity of α-glucosidase (IC_50_ ≥ 1.42 mg/mL)	Not identified	
*T. bellirica* (Gaertn.) Roxb.	Preventive effect on obesity, insulin resistance, and hyperlipidemia in obese type-2 diabetic mice	(3) Gallic acid	(Makihara et al., 2012)
*T. bellirica* (Gaertn.) Roxb.	Reduction in plasma glucose levels, potentiation of insulin secretion from β-cells	(58) Octyl gallate	(Latha and Daisy, 2013)

### Anticancer Studies of *Terminalia* sp.

The World Health Organization states that “cancer is a large group of diseases that can start in almost any organ when abnormal cells grow uncontrollably” (World Health Organization, 2019). Cancer is a non-communicable disease and the second leading cause of death worldwide, only after cardiovascular diseases. In 2018, around 9.6 million people died from cancer, and up to 300,000 new cases are registered per year. Moreover, cancer causes a big economic burden on patients and health care systems with an estimated world annual cost of US$ 1.16 trillion in 2010 (World Health Organization, 2019). Unfortunately, due to the high cost of cancer treatment, people from low and middle-income countries cannot afford conventional drugs and sometimes turn to the use of medicinal plants or extracts from plants for their treatment. Additionally, several chemotherapy agents that are currently used are of plant origin. Thus, studies are still evaluating natural products from new plant species searching for potential anticancer agents. Recent information from the Scopus and Web of Science databases (2010–2020) indicates an ongoing interest in evaluating the anticancer potential of *Terminalia* species.

Wang et al. (2015), evaluated the antiproliferative potential of aqueous extracts of *Terminalia chebula* Retz. using human lung cancer A and mouse lung cancer LLC cell lines. This report isolated five different fractions from the aqueous extracts and shows that *T. chebula* Retz. extracts inhibit cell proliferation by inducing apoptosis and cell-cycle arrest by regulating the mitochondrial pathway mediated by proteins of the Bcl-2 family, inducing the PARP cleavage, and promoting cytochrome c release into the cytoplasm. *T. ferdinandiana* Exell from Australia was studied by Shalom and Cock (2018); the authors evaluated methanolic, aqueous, ethyl acetate, chloroform, and hexane extracts. It was shown that all fruit extracts significantly had an inhibitory effect against Caco-2 cells; though, only methanol and aqueous fruit extracts showed HeLa antiproliferative activity. The antiproliferative potential as determined in IC_50_ values showed that the most potent extracts were ethyl acetate (IC_50_ = 6 µg/mL) against MC3T3-E1 cells, methanolic extracts against MC3T3-E1 (IC_50_ = 40 µg/mL), and Jeg-3 (IC_50_ = 147 µg/mL) cells. As ethyl acetate extracts were the most antiproliferative, the authors only evaluated the phytochemical composition of these samples. It was revealed that this extract is rich in (5) corilagin, (48) luteolin, and (76) chebulic acid. Further studies showed that the mechanism of action was through elevation of the caspase-3 activity, indicating an apoptosis-induced effect.

(58) Octyl gallate and (3) gallic acid isolated from *T. bellirica* (Gaertn.) Roxb. had an antiproliferative effect against MCF-7 (IC_50_ = 40 µg/mL) and MDA-MB-231 cell lines through induced apoptosis by altering the expression of the cell regulators cyclin D1, D3, CDK-4, CDK-6, p18 INK4, p21Waf-1, and p27 KIP. The extracts downregulated the overexpressed cyclin D/CDKs, molecules involved in the progression of the cell cycle through G1 to S phase, which contributes to the induction of apoptosis. Moreover, docking studies confirmed that (58) octyl gallate and (3) gallic acid have a strong binding affinity with the cell cycle regulators by hydrogen bonds. Another group of compounds with antiproliferative potential is furfuran lignans from *T. citrina* (Gaertn.) Roxb. Muhit et al. (2016), tested these furfuran lignans at concentrations of 0.01, 0.1, 1.0, and 10 µM, and the compounds (77) terminaloside B and (78) terminaloside G exhibited antiproliferative effect for MCF-7 and T47D cell lines with suppression of nearly 90% at concentrations lower than 10 µM. Moreover, (79) 6-epiterminaloside K and (80) terminaloside C, (81) terminaloside F, and (82) terminaloside I showed antiestrogenic activity against MCF-7 cells. The antiproliferative potential of these compounds was mainly attributed to the metabolism of the gut microbiota, which produces mammalian lignan metabolites with estrogenic activity like (83) enterodiol and (84) enterolactone (Muhit et al., 2016).

Also, leaf ethanol extracts of *T. catappa* L. from Taiwan was reported by Lee et al. (2019), who used HeLa and SiHa cervical cancer cell lines to test its antiproliferative effect. The authors evaluated *Terminalia* sp. extracts at a concentration of 25, 50, 75, and 100 µg/mL. It was shown that *Terminalia* sp. extracts have low cytotoxicity and suppress TPA-induced migration and invasion through the inhibition of MMP-9 and ERK1/2 phosphorylation in the cell lines in a dose-dependent manner (Lee et al., 2019). Likewise, water extracts from *T. chebula* Retz. from the Republic of Korea reported by Lee et al. (2016), showed antiproliferative potential in HeLa cell lines. The authors state that *T. chebula* Retz. extracts antagonize with the production of mitochondrial-derived reactive oxygen species, which may be related to the author’s hypothesis that due to the previously reported antioxidant activity of *T. chebula* Retz. This extract may have a role in the inhibition of TNF-induced necroptotic cell death. Moreover, HPLC and UHPLC-MS analyses exhibited that water extracts from *T. chebula* Retz. were rich in (3) gallic acid (553.79 nmol/mg), (85) geraniin (80.78 nmol/mg), (76) chebulic acid (54.60 nmol/mg), (86) punicalagin (10.48 nmol/mg), (9) chebulinic acid (10.32 nmol/mg), and (53) chebulagic acid (9.24 nmol/mg). These compounds may be related to its necroptotic cell death induction activity (Lee et al., 2016).

Another report attributes the antiproliferative activity of *T. chebula* Retz. to (53) chebulagic acid in a dose-dependent manner in retinoblastoma cells. The mode of action was suggested through modulation of MMP, induction of the release of cytochrome c, activated caspase 3, and modulated ratio of BAX and Bcl2 in cell death (Kumar et al., 2014). Kumar et al. (2014), treated Y79 cells with different (53) chebulagic acid concentrations (0.001, 0.01, 0.1, 0.5, 1, 5, 10, 25, 50, and 100 µM), which decreased the proliferation of the cells in a dose-dependent manner. Only at a concentration of 50 µM of (53) chebulagic acid, a 50% antiproliferative effect was observed, which was attributed to the capacity of (53) chebulagic acid to induce G1 arrest, inhibit NFκB and induce apoptosis in retinoblastoma Y79 cell lines by induction of the release of cytochrome c by modulating the mitochondrial membrane potential and altering BAX/Bcl2 ratio. Also, *Terminalia* sp. extracts have shown antiproliferative potential against breast cancer, as it is reported by Ghate et al. (2014). The authors showed that methanolic extracts from *T. bellerica* (Gaertn.) Roxb. at a concentration of 100 µg/mL had antiproliferative activity against human breast MCF-7 and human lung A549 carcinoma cell lines. *Terminalia* sp. extracts induced apoptosis by affecting the Bax/Bcl-2 ratio (proapoptotic and antiapoptotic proteins, respectively) in both cell types. HPLC analysis of *T. bellerica* (Gaertn.) Roxb. fruits methanolic extracts exhibited (35) quercetin, (3) gallic acid, (87) catechin, (88) reserpine, and (89) tannic acid as potential active constituents. A Summary of the anticancer studies with different *Terminalia* species is presented in **Table 4**. In addition, **Figure 4(ii)** shows the structures of some of the active constituents with potential antiproliferative and anticancer properties of *Terminalia* species.

**Table 4:** Summarization of the anticancer studies with different *Terminalia* species.

*Terminalia* sp.	Mechanism/Mode of action	Phytochemicals (compound no.)	Reference
*T. chebula* Retz.	Inhibition of cell proliferation by induction of apoptosis and cell-cycle arrest by regulation of the Bcl-2 family	Not identified	(Wang et al., 2015)
*T. ferdinandiana* Exell	Antiproliferative activity against carcinoma cell proliferation	Ethyl acetate extracts: (5) corilagin, (48) luteolin, (76) chebulic acid, (90) protocatechuic acid, (118) rutin, (91) punicalin, (53) chebulagic acid	(Shalom and Cock, 2018)
*T. citrina* (Gaertn.) Roxb.	Antiproliferative and antiestrogenic activity against MCF-7 cells; antiproliferative activity against T47D cell lines	(91) Terminaloside A, (77) terminaloside B, (80) terminaloside C, (92) terminaloside D, (93) terminaloside E, (81) terminaloside F, (78) terminaloside G, (94) terminaloside H, (82) terminaloside I, (95) terminaloside J, (96) terminaloside K, (97) 2-epiterminaloside D, and (79) 6-epiterminaloside K	(Muhit et al., 2016)
*T. catappa* L.	Inhibition of cellular migration and invasion in human HeLa and SiHa cervical cancer cell lines	Not identified	(Lee et al., 2018)
*T. chebula* Retz.		(86) Punicalagin, (85) geraniin, (76) chebulic acid, (9) chebulinic acid, (3) gallic acid	(Lee et al., 2016)
*T. chebula* Retz.	Inhibition of the TNF-induced necroptotic cell death	(3) Gallic acid (hypothesized active compound), (85) geraniin, (76) chebulic acid, (86) punicalagin, (9) chebulinic acid, (53) chebulagic acid	
*T. chebula* Retz.	Chebulagic acid induces G1 arrest and induces apoptosis in retinoblastoma Y79 cells	(53) Chebulagic acid	(Kumar et al., 2014)
*T. bellerica* (Gaertn.) Roxb.	Induced apoptosis in human lung A459 and human breast MCF-7 cancer cell lines	(35) Quercetin, (3) gallic acid, (87) catechin, (89) tannic acid, (88) reserpine	(Ghate et al., 2014)

### Antiinflammatory Properties of *Terminalia* sp.

Inflammation plays a key role in many human diseases. Recent studies show that many noncommunicable diseases share common pathophysiological mechanisms, where oxidative stress and inflammation play a major role in the onset and development of these diseases (Camps and García-Heredia, 2014). Inflammation has been related to obesity, diabetes, cancer, cardiovascular diseases, among others, through various mechanisms (Ghosh et al., 2015). Thus, many investigations have focused on the antiinflammatory pharmaceutical potential of phytochemicals and natural products, aiming to ameliorate adverse effects from antiinflammatory drugs (Ambriz-Pérez et al., 2016). In this sense, some *Terminalia* species have been reported with antiinflammatory activity *in vitro*, which is regularly attributed to their phytochemical composition; the association between the antiinflammatory activity and the structural characteristics of some phytochemicals has already been reported (Gautam and Jachak, 2009; Lago et al., 2014). In this section, we summarize some of the most recent reports on the antiinflammatory potential of *Terminalia* species.

Khan et al. (2018), evaluated the anti-inflammatory capacity of *Terminalia coriacea* (Roxb.) Wight & Arn. (*Terminalia coriacea* Spreng.), in albino Wistar rats with an acute and chronic model, carrageenan-induced paw edema and cotton pellet-induced granuloma, respectively. *T. corriacea* (Roxb.) Wight & Arn. was evaluated at different concentrations (125, 250, and 500 mg/kg) by oral administration of the leaf extract. All tested concentrations showed antiinflammatory activity at a dose-dependent manner, which the authors attribute to the antioxidant potential of *Terminalia* sp. flavonoids like (97) apigenin, (36) kaempferol, (48) luteolin, (98) myricetin, (35) quercetin, and (118) rutin; which was reflected as a decreased paw volume and wet and dry weights of granulomatous tissue in both models of inflammation (Khan et al., 2018).

*Terminalia chebula* Retz. fruit ethanolic extract was evaluated at concentrations of 50 to 500 mg/kg, p.o. against carrageenan-induced inflammation in rats. In this study, Bag et al. (2013), report an increased inhibitory potential on carrageenan-induced lipid peroxidation in rat liver in a dose-dependent manner, with the highest inhibition (84.08%) at 250 mg/kg, p.o.). A study by Reddy et al. (2010), evaluated the COX-2 inhibitory capacity of isolated (3) gallic acid from the ethanolic extract of *Terminalia bellirica* (Gaertn.) Roxb. fruits obtained by RP-HPLC. The authors report that (3) gallic acid has a concentration-dependent inhibitory capacity of COX-1 and COX-2, with IC_50_ values of 1.5 µM and 74 µM, respectively. Further biochemical tests showed competitive binding of (3) gallic acid for both COX-1 and COX-2 concerning substrate, and a time-dependent inhibition for both molecular targets. It is also reported that (3) gallic acid, binding to COX-2 is mediated *via* the carboxylate moiety of (3) gallic acid with the amino acids Arg120 and Glu524 at the entrance of the active site.

Dawe et al. (2017), identified and isolated 11 triterpenes from ethyl acetate extracts of the root bark of *Terminalia glaucescens* Planch. ex Benth.; the compounds were identified as (99) termiglaucescin; (100) β-D-glucopyranosyl 2α, 3β, 6β-trihydroxy-23-gallylean-12-en-28-oate; (101) arjunglucoside I; (102) sericoside; (103) arjungenin; (104) sericic acid; (105) arjunetin; (106) chebuloside II; (107) 3,3’4-tri-O-methylelagic acid; (108) 3,3’-di-O-methylelagic acid; (37) β-sitosterol; and (10) stigmasterol. Moreover, these compounds also showed anti-inflammatory activity by inhibition of albumin denaturation and hemolysis (Dawe et al., 2017). Furthermore, the anti-inflammatory activity of *Terminalia muelleri* Benth. polyphenol-rich extracts were determined by Fahmy et al. (2017), using carrageenan-induced paw edema model in mice by measuring the thickness of the injected paws after treatment with *Terminalia* sp. extract at a concentration of 100, 200, and 400 mg/kg. Pretreatment with *Terminalia* sp. extracts exhibited a dose-dependent significant anti-inflammatory activity showed in the reduction in the carrageenan-induced paw edema by 48, 53, and 62% at 100, 200, and 400 mg/kg, respectively. Moreover, pretreatment with *Terminalia* sp. extracts also decreased in a dose-dependent manner, the pro-inflammatory cytokines PGE2, TNF-α, IL-1β, and IL-6. The authors suggested that this effect might be attributed to the presence of (109) luteolin-6-C-glucoside, (110) vitexin, (4) ellagic acid, and (9) chebulinic acid in the extracts (Fahmy et al., 2017).

Crude extracts from *Terminalia phanerophlebia* Engl. & Diels, a species endemic to Africa, exhibit selective inhibition of COX-2 (92.4%). The bioactivity was attributed to the cholestane triterpenoids such as (37) β-sitosterol, (111) β-sitostenone, and (112) stigmast-4-ene-3,6-dione; which were further isolated and individually evaluated, this exhibited that the COX-2 inhibitory activity might be attributed to the triterpenoid (37) β-sitosterol (Nair et al., 2012). Similarly, *Terminalia bellirica* (Gaertn.) Roxb. ethyl acetate from the aerial parts at concentrations of 100 and 300 mg/kg was orally administered to male BALB/cN mice following tetrachloride-intoxication. *Terminalia* sp. treatment downregulated the expression of the inflammatory mediators NF-κB, COX-2, and TNF-α. A summary of the antiinflammatory studies of different *Terminalia* species is presented in **Table 5**. The chemical structures of some bioactive compounds from *Terminalia* species with antiinflammatory potential are shown in **Figure 4(iii)**.

**Table 5:** Summarization of the antiinflammatory studies of different *Terminalia* species.

*Terminalia* sp.	Mechanism/Mode of action	Phytochemicals (compound no.)	Reference
*T. coriacea* (Roxb.) Wight & Arn.	Decreased paw volume and wet and dry weights of granulomatous tissue in acute and chronic models of inflammation in rats	(97) Apigenin, (36) kaempferol, (48) luteolin, (98) myricetin, (35) quercetin, and (118) rutin	(Khan et al., 2018)
*T. bellerica* (Gaertn.) Roxb.	Selective binding of gallic acid with the amino acids Arg120 and Glu524 from COX-2	(3) Gallic acid	(Reddy et al., 2010)
*T. glaucescens* Planch. ex Benth.	Inhibition of albumin denaturation and hemolysis	(99) termiglaucescin; (100) β-D-glucopyranosyl 2α, 3β, 6β-trihydroxy-23-gallylean-12-en-28-oate; (101) arjunglucoside I; (102) sericoside; (103) arjungenin; (104) sricic acid; (105) arjunetin; (106) chebuloside II; (107) 3,3’4-tri-O-methylelagic acid; (108) 3,3’-di-O-methylelagic acid; (37) β-sitosterol; and (10) stigmasterol	(Dawe et al., 2017)
*T. muelleri* Benth.	Reduction of paw edema in carrageenan-induced paw edema in mice. Reduced proinflammatory cytokines PGE2, TNF-α, IL-1β, and IL-6	(109) luteolin-6-C-glucoside, (110) vitexin, (4) ellagic acid, and (9) chebulinic acid	(Fahmy et al., 2017)

*Antioxidant Potential of Terminalia* sp.

Oxidative stress is frequently defined as the imbalance between antioxidants and oxidants in favor of the oxidants, and this may lead to oxidative damage to molecules of biological importance (Halliwell and Gutteridge, 2015). Oxidative stress main arises from diverse factors such as diminished levels of antioxidant enzymes, increased production of reactive species (due to unhealthy lifestyles, smoking, abusive intake of alcohol, among others). Oxidative stress has been related to the onset of many diseases and their comorbidities (Camps and García-Heredia, 2014; Halliwell and Gutteridge, 2015). *Terminalia* species are a rich source of phytochemicals such as terpenes, flavonoids, and phenolic acids, molecules with reported antioxidant activity. This section summarizes some of the most recent publications on this subject.

Anokwuru et al. (2018), evaluated the antioxidant capacity of ethanolic extracts of fruit, leaf, stem, bark, and roots of *Terminalia sericea* Burch. ex DC. from South Africa; moreover, free, conjugated, and bound phenolic-rich extracts were obtained. The antioxidant activity was evaluated by the DPPH method. The authors reported that the free fruit extracts, ester bound of leaves and roots, glycoside bound fruit extracts, leaves, and stem insoluble bound extracts showed the highest antioxidant activity with IC_50_ values of 3.13, 4.58, and 4.89, 12.6, 15.4, and 17.8 µg/mL, respectively. Furthermore, the antioxidant activity was attributed mainly to the presence of (3) gallic acid and (113) resveratrol. Another evaluation of polyphenolic-rich extracts was performed by Saha and Verma (2016), but with *Terminalia chebula* Retz. fruit methanolic extracts at concentrations ranging from 50–500 µg/mL; the authors evaluated the antioxidant capacity of the samples by different methods. The total antioxidant capacity assay showed that *T. chebula* Retz. extracts had the highest antioxidant potential, in a dose-dependent manner, at a concentration of 150 µg/mL yielding an IC_50_ value of 14 µg/mL. Moreover, *T. chebula* Retz. extracts also inhibited nitric oxide with an IC_50_ value of 30.51 µg/mL at a concentration of 500 µg/mL; the extracts also showed scavenging activity towards H_2_O_2_, which is not a free radical but the precursor of some reactive oxygen species, with an IC_50_ value of 265.53 µg/mL.

Studies on *Terminalia chebula* Retz. collected in India and extracts prepared from fruits with 70% ethanol were reported by Bag et al. (2013). The antioxidant capacity was measured by the liver lipid peroxidation and DPPH assays; *T. chebula* Retz. extracts exhibited a dose-dependent antioxidant capacity with the highest inhibition of the DPPH radical at a concentration of 50 µg/mL with 58.40% antioxidant percentage and IC_50_ of 42.14 µg/mL. Moreover, the extracts also reduced the formation of MDA in a dose-dependent manner, with the highest activity at a dose of 250 mg/kg p.o.

Methanolic extracts of fruits of *T. bellerica* (Gaertn.) Roxb. from India were studied by Basu et al. (2017). The extracts were further sequentially extracted with n-hexane, chloroform, ethyl acetate, butanol, and water to obtain different fractions. The obtained extracts were evaluated by the Trolox Equivalent Antioxidant Capacity method, and the results showed that the butanol, water, and ethyl acetate extracts had the highest antioxidant capacity. Moreover, the ethyl acetate extracts showed the highest DPPH scavenging radical activity with an IC_50_ value of 7.11 µg/mL. The authors mention that the polar fractions showed higher antioxidant activity might be related to the presence of compounds like (114) purpurin, (87) catechin, (89) tannic acid, (88) reserpine, (4) ellagic acid, (70) methyl gallate, and (118) rutin (Basu et al., 2017). Furthermore, Dawe et al. (2017), showed that ethanolic extracts from roots of *T. glaucescens* Planch. ex Benth. have scavenging activity against the DPPH radical, which may be attributed to the compounds (99) termiglaucescin; (100) β-D-glucopyranosyl 2α, 3β, 6β-trihydroxy-23-gallylean-12-en-28-oate; (101) arjunglucoside I; (102) sericoside; (103) arjungenin; (104) sericic acid; (105) arjunetin; (106) chebuloside II; (107) 3,3’4-tri-O-methylelagic acid; (108) 3,3’-di-O-methylelagic acid; (37) β-sitosterol; and (10) stigmasterol (Dawe et al., 2017).

*Terminalia chebula* Retz. fruit extracts were tested for their antioxidant and antiinflammatory properties on acetic acid-induced colitis in inbred Charles-Foster strain albino rats and mice by Gautam et al. (2013). This report states that *T. chebula* Retz. extracts reversed the decreased levels of superoxide dismutase, catalase, and glutathione peroxidase caused by acetic acid-induced colitis, *Terminalia* sp. treatment also decreased the levels of lipid peroxidation and nitric oxide to normal levels, from 11 to 6.22, and 10 to 4.25 nmol/mg protein, respectively. A preliminary phytochemical screening test showed that the main constituents in *T. chebula* Retz. extracts are tannins, phenolic compounds, and triterpenoids; however, further characterization is needed (Gautam et al., 2013).

Furthermore, Rashed et al. (2014), evaluated the antioxidant capacity of extracts from the aerial parts of *T. bellerica* (Gaertn.) Roxb. on carbon tetrachloride-intoxicated mice. Preliminary antioxidant assays by the DPPH and ABTS tests showed that ethanolic extracts had a higher antioxidant activity with values of 2883.38 and 2414.81 TEAC/mg against the radicals, respectively. Moreover, *in vivo* studies showed that *T. bellerica* (Gaertn.) Roxb. extracts at a concentration of 300 mg/kg ameliorated in a dose-dependent manner, the production of 4-hydroxynonenal and 3-nitrotyrosine (Rashed et al., 2014). *Terminalia chebula* Retz. plant extracts obtained by ultrasonic-assisted extraction optimized using response surface methodology were reported by Sheng et al. (2018). The authors report the optimal factors for phenolic extraction of *T. chebula* Retz. were 68% ethanol concentration, the ultrasonic intensity of 3.6 W/cm^2^, the particle size of 0.18 mm, extraction temperature and time of 20 min for 2 times at 70°C, and the liquid-solid ratio of 23 mg/mL. Optimized results yield higher DPPH and ABTS antioxidant capacity in a dose-dependent manner, ultrasound-assisted extracts showed higher antioxidant activity than ascorbic acid by the FRAP method at 0.010-0.013 mg/mL, and by the DPPH method at concentrations from 0.003–0.011 mg/mL. An HPLC-DAD-ESI-MS analysis showed the presence of (114) shikimic acid; (3) gallic acid; (115) 5-O-galloylshikimic acid; (5) corilagin; (116) 3,4,8,9,10-pentahydroxydibenzo (b,d) pyran-6-one; and (4) ellagic acid, could be responsible for the antioxidant properties of *T. chebula* Retz. fruit extracts (Sheng et al., 2018). A summary of the antioxidant potential of different *Terminalia* species is shown in **Table 6**. The chemical structures of some bioactive compounds from *Terminalia* species with antioxidant potential is shown in **Figure 4(iv)**.

**Table 6:** Summarization of the antioxidant studies of different *Terminalia* species.

*Terminalia* sp.	Mechanism/Mode of action	Phytochemicals (compound no.)	Reference
*T. sericea* Burch. ex DC.	Inhibition of the DPPH radical	(3) gallic acid and (113) resveratrol	(Anokwuru et al., 2018)
*T. chebula* Retz.	Scavenging activity towards the DPPH radical, nitric oxide and H_2_O_2_	Not identified	(Saha and Verma, 2016)
*T. chebula* Retz.	Scavenging activity against the DPPH radical	Not identified	(Bag et al., 2013)
*T. bellerica* (Gaertn.) Roxb.	Scavenging activity against the ABTS and DPPH radicals	(114) purpurin, (87) catechin, (89) tannic acid, (88) reserpine, (4) ellagic acid, (70) methyl gallate, and (118) rutin	(Basu et al., 2017)
*T. chebula* Retz.	Normalized levels of lipid peroxidation, nitric oxide, superoxide dismutase, glutathione peroxidase, and catalase in rats with acetic acid-induced colitis	Tannins, triterpenoids, phenolic compounds	(Gautam et al., 2013)
*T. chebula* Retz.	Optimized ultrasound-assisted extraction of ethanol extracts yields compounds with DPPH and ABTS scavenging activity	(114) shikimic acid; (3) gallic acid; (115) 5-O-galloylshikimic acid; (5) corilagin; (116) 3,4,8,9,10-pentahydroxydibenzo (b,d) pyran-6-one; and (4) ellagic acid	(Sheng et al., 2018)

### Antimicrobial Properties of *Terminalia* sp.

*Terminalia* species are documented with ethnobotanical used against infectious diseases/ailments such as conjunctivitis, diarrhea, dysentery, pneumonia, flu, and sore throats, sexually transmitted diseases, urinary infections, among others (Eloff et al., 2008; Maulik and Katiyar, 2010; Maulik and Talwar, 2012; Cock, 2015; Afshari et al., 2016). This has led to some studies as an effort to elucidate their antimicrobial mechanisms and antimicrobial spectrum. Some efforts are being made to evaluate the potential antimicrobial properties of *Terminalia* sp. extracts. The report by Akter et al. (2019), evaluates the antimicrobial capacity of *Terminalia ferdinandiana* Exell. extracts in food preservation. The authors prepared the extracts with methanol, ethanol, acetone, hexane, and distilled water, by accelerated solvent extraction from freeze-dried powders of barks, fruits, and leaves of *T. ferdinandiana* Exell, and tested them against some of the most common foodborne microorganisms by the disc diffusion assay. The authors found that methanol extracts showed a broad spectrum of antibacterial activity against the gram-positive *Staphylococcus aureus*, methicillin-resistant *Staphylococcus aureus, Bacillus cereus, Listeria monocytogenes*, and the gram-negative bacteria *Pseudomonas aeruginosa*. Also, the authors determined that the minimum inhibitory concentration and minimum bactericidal concentration values of extracts of *T. ferdinandiana* Exell ranged from 1–3 mg/mL, *L. monocytogenes*, *B. cereus*, and methicillin-resistant *S. aureus* were the most sensitive bacteria against *Terminalia* sp. extracts; on this subject, ethanol and acetone extracts showed the most potent antibacterial inhibitory activity. The authors argue that intriguingly, the extracts with the highest antioxidant activity (methanol and water extracts) were not the ones with the highest antibacterial activity, which may indicate that the compounds that may be differentially found in each type of extracts (Akter et al., 2019).

Moreover, *T. bellirica* (Gaertn.) Roxb. dried fruits were used to obtain direct and sequential dichloromethane, methanol, and water extracts, and to evaluate their antibacterial activity against 16 strains of methicillin-resistant *Staphylococcus aureus*, spectrum β-lactamase producing *Escherichia coli*, and methicillin-resistant *Acinetobacter* sp., *Klebsiella pneumoniae*, and *Pseudomonas aureginosa* (Dharmaratne et al., 2018). The minimum inhibitory concentration values showed that all aqueous and methanol extracts have antibacterial activity with values ranging from 0.25 to 4 mg/mL against all strains tested, which indicates that further studies are needed to test the antimicrobial potential of *Terminalia* sp. extracts before designing broad-spectrum antibacterial drugs based of *Terminalia* sp. (Dharmaratne et al., 2018)

*T. ferdinandiana* Exell has also been studied for their potential antibacterial properties to evaluate their inhibitory capacity against odor-forming bacteria like *Corynebacterium jeikeium, Staphylococcus epidermidis, Propionibacterium acnes*, and *Brevibacterium linens* (Mcmanus et al., 2017). Methanolic extracts from leaves of *T. ferdinandiana* Exell showed the lowest minimum inhibitory capacity values against *C. jeikeium* (233 µg/mL), *S. epidermidis* (220 µg/mL), *P. acnes* (625 µg/mL), and B linens (523 µg/mL). Moreover, Mcmanus et al. (2017) determined that leaf extracts of *T. ferdinandiana* Exell were non-toxic by the *Artemia franciscana* bioassay, interestingly; chloroform and hexane fruit and leaves extracts showed no toxicity activity. LC-MS analysis showed that methanolic extracts of *T. ferdinandiana* Exell contained some tannins and other compounds, the most abundant were (117) ellagic acid dihydrate, (76) chebulic acid, (4) ellagic acid, (44) castalagin, (5) corilagin, (91) punicalin, (48) luteolin, (9) chebulinic acid, (119) exifone, (53) chebulagic acid, and (120) trimethyl ellagic acid (Mcmanus et al., 2017).

Lee et al. (2017), reported the potential use of ethanol extracts from fruits of *Terminalia chebula* Retz. to prevent dental plaque bacteria-mediated periodontal disease for their *Streptococcus mutans* and *Aggregatibacter actinomycetemcomitans* growth-inhibitory capacity. Moreover, concomitant with the anti-inflammatory activity of *T. chebula* Retz. extracts, as exhibited by their inhibitory capacity of PGE2 and COX-2. A mixture prepared with 5 mg/mL *A. malaccensis* and 20 mg/mL *T. catappa* L. was evaluated for its antimicrobial properties against *L. monocytogenes* *and S. aureus*, in vacuum packed ready-to-cook chicken, inhibited the growth of *S. aureus* with 1.80, 2.13, 2.36, and 2.97 log CFU/g reduction in 3,6,9, and 12 days, and also decreased the growth of *L. monocytogenes* with 1.22, 1.60, and 1.55 log CFU reduction at 6,9, and 12 days, respectively. These results may indicate the ability of *A. malaccensis* and *T. catappa L.* extracts to extend shelf-life of chicken meat in vacuum packed ready-to-cook chicken (Somarathna et al., 2018).

*Terminalia* sp. extracts have been the center of studies regarding the biogenic synthesis of nanoparticles with therapeutic potential. For instance, Sivamaruthi et al. (2019), aimed to biosynthesize silver Palladium bimetallic nanoparticles from aqueous fruit extract of *Terminalia chebula* Retz., as potential antimicrobial and anticancer agents. The nanoparticles exhibited antimicrobial activity against gram-positive bacterial strains like methicillin-resistant *S. aureus* MSRA 11 and MRSA56; and gram-negative bacteria *P. aeruginosa* with a zone of inhibition from 12–16 mM at a concentration of 30 µg/mL. Further toxicity studies showed no cytotoxicity in peripheral blood mononuclear cells, even at the highest dose of nanoparticles of 200 µg/mL (Sivamaruthi et al., 2019). Akhter et al. (2019), evaluated the antibacterial activity of biogenic synthesized nanoparticles of zinc, copper, and iron oxides using the extract of *Terminalia bellirica* (Gaertn.) Roxb. fruits, against gram-positive *Staphylococcus aureus*, and gram-negative *Bacillus subtilis, Escheerichia coli, Klebsiella pneumoniae*, and *Salmonella enterica*. The zone of inhibition of the evaluated nanoparticles exhibited its maximum values for zinc oxide nanoparticles.

Furthermore, studies using *T. bellirica* (Gaertn.) Roxb. fruit extracts to biosynthesize gold nanoparticles have been reported by Annavaram et al. (2017), who evaluated their antifungal potential. The authors showed that gold nanoparticles against *Candida tropicalis* and *Candida albicans* exhibited maximum zone of inhibition of around 16 and 14 mm, respectively. This effect was partially attributed to the presence of (3) gallic acid, (4) ellagic acid, (70) methyl gallate, (37) β-sitosterol, and (53) chebulagic acid. **Figure 4(v)**, shows the chemical structure of some important compounds with antimicrobial properties.

#### Antibacterial Activity of *Terminalia* sp.

As shown in **Table 7**, *Terminalia superba* Engl. & Diels bark extract inhibited diarrhea-causing pathogens including *Shigella dysenteriae* and *Salmonella typhi* (Kuete et al., 2010). The bark, fruit, and seed coat extracts of *Terminalia ferdinandiana* Exell inhibited *Bacillus cereus* and methicillin-resistant *Staphylococcus aureus* with inhibition zone ranging from 7–17.8 mm (Akter et al., 2019). Moreover, the extracts of *Terminalia* sp. members possessed antibacterial activity against airborne pathogens. MDR *Acinetobacter* sp. and the growth of MDR *Pseudomonas aeruginosa* was suppressed when the bacteria were treated with the fruit extract of *Terminalia bellirica* (Gaertn.) Roxb. (Dharmaratne et al., 2018) and *Terminalia chebula* Retz. (Sharma et al., 2012). In addition, *Terminalia glaucescens* Planch. ex Benth. root (Gbala and Anibijuwon, 2018) and *Terminalia superba* Engl. & Diels bark extract (Kuete et al., 2010) inhibited *Klebsiella* *pneumoniae*, a pneumonia-causing agent, with MIC at 0.1 and 0.078 mg/mL, respectively. Interestingly, the bark of *Terminalia superba* Engl. & Diels inhibited *Mycobacterium tuberculosis* with a low MIC value of 0.078 mg/mL (Kuete et al., 2010). This report suggested that extracts from *Terminalia* sp. plant species could be used as antibacterial agents against pathogenic bacteria.

**Table 7:** Antibacterial potential of *Terminalia* sp.

Plant species/part	Extraction procedure	Pathogenic bacteria	Antibacterial activity	Reference
*Terminalia arjuna* (Roxb. ex DC.) Wight & Arn. bark	Ethanol extraction	*B. cereus*	IZ = 7 mm	(Saivaraj and Chandramohan, 2018)
	Ethanol extraction	*P. aeruginosa *	IZ = 8 mm	
	Ethanol extraction	*S aureus*	MIC = 1.56 mg/mL	(Aneja et al., 2012)
	Ethanol extraction	*E. coli*	MIC = 50 mg/mL	
	Ethanol extraction	*P. mirabilis*	MIC = 50 mg/mL	
*Terminalia* *arjuna* (Roxb. ex DC.) Wight & Arn. leaf	Ethanol extraction	*S. aureus*	MIC = 6.25 mg/mL	(Aneja et al., 2012)
	Ethanol extraction	*P. aeruginosa*	MIC = 50 mg/mL	
	Ethanol extraction	*P. mirabilis*	MIC = 6.25 mg/mL	
*Terminalia bellirica* (Gaertn.) Roxb. fruit	Direct aqueous extracts/Reflux method	MRSA	MIC = 0.25 mg/mL	(Dharmaratne et al., 2018)
	Direct aqueous extracts/Reflux method	MDR *Acinetobacter* sp.	MIC = 0.5 mg/mL	
	Direct aqueous extracts/Reflux method	MDR *P. aeruginosa*	MIC = 0.5 mg/mL	
*Terminalia catappa* L. leaf	Ethanol extraction	*B. subtilis*	MIC = 3 mg/mL	(Suparno et al., 2018)
	Ethanol extraction	*E. coli *	MIC = 3 mg/mL	
	Ethanol extraction	*P. aeruginosa*	IZ = 1.83-6.5 mm	(Allyn et al., 2018)
	Ethanol extraction	*S. aureus*	IZ = 1.73-9.06 mm	
*Terminalia chebula* Retz. fruit	Ethanol extraction	*Acinetobacter* sp.	MIC = 12.5 mg/mL	(Sharma et al., 2012)
	Ethanol extraction	*E. coli *	MIC = 50 mg/mL	
	Ethanol extraction	*P. mirabilis*	MIC = 25 mg/mL	
	Ethanol extraction	*P. aeruginosa*	MIC = 12.5 mg/mL	
	Ethanol extraction	*S. aureus*	MIC = 3.12 mg/mL	
*Terminalia ferdinandiana* Exell bark	Ethanol extraction	*B. cereus*	IZ = 13.2 mm	(Akter et al., 2019)
	Ethanol extraction	MRSA* *	IZ = 12.7 mm	
*Terminalia ferdinandiana* Exell fruit	Ethanol extraction	*B. cereus*	IZ = 17.8 mm	
	Ethanol extraction	*L. monocytogenes*	IZ = 18.5 mm	
	Ethanol extraction	MRSA	IZ = 17.1 mm	
*Terminalia ferdinandiana* Exell seed coat	Ethanol extraction	*B. cereus*	IZ = 9.8 mm	
	Ethanol extraction	*L. monocytogenes*	IZ = 10.8 mm	
	Methanol extraction	MRSA	IZ = 8.8 mm	
*Terminalia glaucescens* Planch. ex Benth. root	Ethanol extraction	*E. aerogenes*	MIC = 100 mg/mL	(Gbala and Anibijuwon, 2018)
	Ethanol extraction	*K. pneumoniae*	MIC = 0.1 mg/mL	
	Ethanol extraction	*P. mirabilis *	MIC = 100 mg/mL	
*Terminalia superba* Engl. & Diels bark	Methanol extraction	*E. coli*	MIC = 0.078 mg/mL	(Kuete et al., 2010)
	Methanol extraction	*K. pneumoniae*	MIC = 0.078 mg/mL	
	Methanol extraction	*M. tuberculosis*	MIC = 0.078 mg/mL	
	Methanol extraction	*P. aeruginosa *	MIC = 0.019 mg/mL	
	Methanol extraction	*S. dysenteriae *	MIC = 0.039 mg/mL	
	Methanol extraction	*S. typhi *	MIC = 0.078 mg/mL	
	Ethanol extraction	*S. aureus*	MIC = 0.078 mg/mL	(Elvire et al., 2018)

IZ, inhibition zone.

#### Antifungal Activity of *Terminalia* sp.

Filamentous fungi such as *Aspergillus flavus*, *A. niger*, and *Trichophyton rubrum* are some of the important human pathogens. Besides, opportunistic yeast fungi are an important cause of morbidity and mortality in immunocompromised patients. As shown in **Table 8**, the growth of filamentous fungi, including *A. flavus* and *A. niger* was inhibited by *Terminalia glaucescens* Planch. ex Benth. stem extract (Adeeyo et al., 2018). Kuete and his team have shown that the extract from the bark of *Terminalia superba* Engl. & Diels possessed antifungal activity against dermatophytes. The MIC values of the extract against *Microsporum audouinii* and *Trichophyton rubrum* were 0.019 and 0.039 mg/mL respectively (Kuete et al., 2010). *Terminalia arjuna* (Roxb. ex DC.) Wight & Arn. (Saivaraj and Chandramohan, 2018) and *Terminalia catappa* L. (Gonçalves et al., 2019) bark, and *Terminalia chebula* Retz. fruit (Vidya et al., 2019) extract demonstrated antifungal activity against the opportunistic yeasts including *Candida albicans*, *C. glabrata*, *C. krusei*, and *C. tropicalis*. Kouassi et al. (2019) reported that *Terminalia ivorensis* A.Chev. and *Terminalia mantaly* H. Perrier bark extracts inhibited *Fusarium* ssp., plant pathogenic fungi, with low MIC values ranging from 0.025–0.050 mg/mL (Kouassi et al., 2019). Moreover, the leaf extract from *Terminalia catappa* L. exhibited antibiofilm activity against *C. albicans* and *C. glabrata* (Gonçalves et al., 2019). The information indicated that the extracts of *Terminalia* sp. plant species could be used as antifungal agents against pathogenic fungi.

**Table 8:** Antifungal activity of *Terminalia* sp. against pathogenic fungi.

Plant species/part	Extraction Procedure	Pathogenic fungi	Antifungal activity	Reference
*Terminalia arjuna* (Roxb. ex DC.) Wight & Arn. bark	Ethanol extraction	*A. flavus*	IZ = 7 mm	(Saivaraj and Chandramohan, 2018)
	Ethanol extraction	*C. albicans*	IZ = 15 mm	(Debnath et al., 2013)
	Ethanol extraction	*C. glabrata*	IZ = 20 mm	
	Ethanol extraction	*C. krusei*	IZ = 20 mm	
	Ethanol extraction	*C. tropicalis*	IZ = 18 mm	
*Terminalia catappa L.* bark	Ethanol extraction/Methanol extraction	*F. oxysporum*	MIC = 0.025 mg/mL	(Kouassi et al., 2019)
*Terminalia catappa L.* leaf	Ethanol extraction	*C. albicans*	MIC = 0.25 mg/mL Biofilm inhibition	(Gonçalves et al., 2019)
	Ethanol extraction	*C. glabrata*	MIC = 0.25 mg/mL Biofilm inhibition	
	Ethanol extraction	*C. krusei*	MIC = 1.5 mg/mL	
	Ethanol extraction	*C. tropicalis*	MIC = 1.5 mg/mL	
*Terminalia chebula* Retz. fruit	Ethanol extraction	*C. albicans*	MIC = 0.25 mg/mL	(Vidya et al., 2019)
	Ethanol extraction	*C. glabrata*	MIC = 0.25 mg/mL	
	Ethanol extraction	*C. tropicalis*	MIC = 0.050 mg/mL	
*Terminalia glaucescens* Planch. ex Benth. stem	Ethanol extraction	*A. flavus*	IZ = 12.0 mm	(Adeeyo et al., 2018)
	Ethanol extraction	*A. niger*	IZ = 11.0 mm	
	Ethanol extraction	*Candida* sp.	IZ = 11.0 mm	
*Terminalia ivorensis* A.Chev. bark	Ethanol extraction	*F. oxysporum*	MIC = 0.050 mg/mL	(Kouassi et al., 2019)
	Ethanol extraction	*F. graminearum*	MIC = 0.025 mg/mL	
*Terminalia mantaly* H. Perrier bark	Ethanol extraction	*F. oxysporum*	MIC = 0.050 mg/mL	(Kouassi et al., 2019)
	Ethanol extraction	*F. graminearum*	MIC = 0.025 mg/mL	
*Terminalia superba* Engl. & Diels bark	Methanol extraction	*C. albicans*	MIC = 0.039 mg/mL	(Kuete et al., 2010)
	Methanol extraction	*C. glabrata*	MIC = 0.078 mg/mL	
	Methanol extraction	*M. audouinii*	MIC = 0.019 mg/mL	
	Methanol extraction	*T. rubrum*	MIC = 0.039 mg/mL	
	Ethanol extraction	*F. oxysporum*	MIC = 0.050 mg/mL	(Kouassi et al., 2019)
	Ethanol extraction	*F. graminearum*	MIC = 0.050 mg/mL	

IZ, inhibition zone.

### Antiparasitic Activity of *Terminalia* sp.

Parasites, including malaria, are the most significant protozoan disease in the world. In 2018, 228 million cases of malaria worldwide were reported by the World Health Organization. In addition, other parasites such as *Haemonchus contortus* and *Trypanosoma brucei* are the main cause of morbidity and mortality in humans. The present study is focused on the antiparasitic activity of *Terminalia* sp. extract against the important parasites (**Table 9**). Camara and team reported antimalaria activity of *Terminalia albida* Scott-Elliot bark extract against *Plasmodium falciparum* with low IC_50_ as 1.5 µg/mL (Camara et al., 2019). Moreover, an increase in the survival rate of mice infected with the *P. berghei* was detected when they were treated with the bark extract of *Terminalia albida* Scott-Elliot (Camara et al., 2019) and *Terminalia avicennioides* Guill. & Perr. (Owoloye et al., 2019). Interestingly, Mbouna and the team reported that different parts of *Terminalia mantaly* H. Perrier inhibited *P. falciparum*. It has been commented that IC_50_ of *Terminalia mantaly* H. Perrier leaf, root, and stem were 2.09, 7.01, and 3.63 µg/mL, respectively (Mbouna et al., 2018). It has been reported that *Terminalia superba* Engl. & Diels bark, root, and stem possess antitrypanosomal activity against *Trypanosoma brucei brucei* with MIC ranging from 0.8-1.6 mg/mL (Antia et al., 2009). Also, the activity of *Terminalia arjuna* (Roxb. ex DC.) Wight & Arn. bark extract against *H. contortus*, barber’s pole worm, ova, and larva has been documented (Bachaya et al., 2009). This study showed that extracts from *Terminalia* sp. plant species could be used as alternative agents for the treatment of parasitic infection.

**Table 9:** Antiparasitic activity of *Terminalia* sp. against pathogenic parasites.

Plant species/part	Extraction Procedure	Pathogenic parasites	Antiparasitic activity	Reference
*Terminalia albida* Scott-Elliot bark	Methanol extraction	*P. falciparum*	IC_50_ = 1.5 µg/mL	(Camara et al., 2019)
	Methanol extraction	*P. berghei*	*In vivo*, *Terminalia albida* Scott-Elliot treatment increased survival rates in *P. berghei*-infected mice.	
*Terminalia arjuna* (Roxb. ex DC.) Wight & Arn. bark	Methanol in a Soxhlet’s apparatus	*H. contortus* ova	IC_50_ = 645.65 µg/mL	(Bachaya et al., 2009)
	Methanol in a Soxhlet’s apparatus	*H. contortus* larva	IC_50_ = 467.74 µg/mL	
*Terminalia avicennioides* Guill. & Perr. bark	Powder form	*P. berghei*	Inhibition of the malaria in infected mice	(Owoloye et al., 2019)
*Terminalia catappa* L.* *fruit	Ethanol extraction	*P. posthuman*	At 60 mg/ml; death time = 28 min	(Ingole et al., 2019)
*Terminalia catappa* L. leaf	Ethanol extraction	*F. cobboldi*	Motility inhibition within 3 h	(Anuracpreeda et al., 2016)
	Butanol extraction	*C. spatiosus*	LC_50_ = 487.17 ppm.	(Minsakorn et al., 2019)
	Ethyl acetate extraction	*P. falciparum*	IC_50_ = 3.05 µg/mL	(Abiodun et al., 2011)
	Ethyl acetate extraction	*T. brucei rhodesiense*	IC_50_ = 7.80 µg/mL	(Abiodun et al., 2012)
*Terminalia ferdinandiana* Exell fruit	Aqueous extraction	*G. duodenalis*	IC_50_ = 140 µg/mL	(Rayan et al., 2015)
*Terminalia ferdinandiana* Exell fruit pulp	–	*G. duodenalis*	LC_50_ = 1,150 µg/mL	(Cock and Rayan, 2020)
*Terminalia mantaly* H. Perrier leaf	Aqueous extraction	*P. falciparum*	IC_50_ = 2.09 µg/mL	(Mbouna et al., 2018)
*Terminalia mantaly* H. Perrier root	Methanol extraction	*P. falciparum*	IC_50_ = 7.01 µg/mL	(Mbouna et al., 2018)
	Methanol extraction	*P. falciparum*	IC_50_ = 10.11 µg/mL	(Mbosso Teinkela et al., 2019)
	Hexane fraction	*Trypanosoma brucei brucei*	IC_50_ = 5.60 µg/mL	
*Terminalia mantaly* H. Perrier stem	Methanol extraction	*P. falciparum*	IC_50_ = 3.63 µg/mL	(Mbouna et al., 2018)
*Terminalia paniculata* Roth root	Ethanol in a Soxhlet’s apparatus	*P. posthuma*	25 mg/ml; death time = 87 min	(Acharyya and Prasenjit Bhuniya, 2019)
*Terminalia superba* Engl. & Diels leaf	Methanol in a Soxhlet’s apparatus	*Trypanosoma brucei brucei*	MIC = 1.6 mg/mL	(Antia et al., 2009)
	Methanol extraction	*P. falciparum*	IC_50_ = 3.38 µg/mL	(Mbouna et al., 2018)

## Nanoparticles Synthesized Using *Terminalia* sp. Extracts: Improvement of the Bio-Activity

In an attempt to improve the bioactivity of medicinal plants, much research has focused on nanoparticles to reduce the size of particles and increase the surface area. Plant extracts are capping agents capable of reducing metal ion, resulting in the formation of nanoparticles with remarkable antibacterial activities (Majoumouo et al., 2019). Silver nanoparticles (AgNPs) using *Terminalia arjuna* (Roxb. ex DC.) Wight & Arn. bark extract (TA-AgNPs) were synthesized eco-friendly. The antibacterial activity of TA-AgNPs against *E. coli* was better than the extract (Ahmed et al., 2017). MIC values of *Terminalia mantaly* H. Perrier extract and *Terminalia mantaly* -AgNPs (TM-AgNPs) against *Haemophilus influenzae* was reported to be 125 and 3.12 µg/mL, respectively. It was noted that the MIC values of the TM-AgNPs were 40 times lower than those of the extract (Majoumouo et al., 2019). Hence, plant-nanoparticle agents are a significant strategy for the treatment of bacterial infection. *Terminalia chebula* Retz. leaf gold nanoparticles (TC-AgNPs) showed antibacterial activity against *S. aureus* and *E. coli*. The prepared TC-AgNPs were used on Nylon fabrics for future applying of medical materials.

Interestingly, Nylon cloth-TC-AgNPs exhibited antibacterial activity against organisms with a strength of 3 to 13 times greater than TC-AgNPs (Rohaeti and Rakhmawati, 2017). Gold nanoparticles using *Terminalia arjuna* (Roxb. ex DC.) Wight & Arn. leaf extract has been reported to induce mitotic cell division and pollen germination. Moreover, the gold nanoparticles showed a non-cytotoxic effect on root tip cells of *Allium cepa* and pollen grains of *Gloriosa superba* (Gopinath et al., 2013). AgNPs synthesized using the polyphenol-rich ethyl acetate fraction of *Terminalia bellirica* (Gaertn.) Roxb. fruit pericarp, exhibited anticancer activity. Nanoparticles at 120 μg/mL have been reported to kill 69.1% of colon cancer cells and 65.2% breast cancer cells. Interestingly, AgNPs did not cause cytotoxic effects against normal cardiac and skeletal muscle cells (Nampoothiri et al., 2018). This information may indicate that the agents of plant-nanoparticles are a pronounced strategy to improve the bio-activity of medicinal plants in the treatment of many diseases. **Table 10** shows the bioactive potential of several types of nanoparticles synthesized using the extracts of *Terminalia* sp.

**Table 10:** Bio-activity of nanoparticles synthesized using *Terminalia* sp. extracts.

Plant species/Part used	Plant-nanoparticle	Bio-activity	References
*Terminalia arjuna* (Roxb. ex DC.) Wight & Arn. bark	Silver nanoparticles (TA-AgNPs)	TA-AgNPs inhibited *E. coli*, while the extract exhibited non-effects on the organism.	(Ahmed et al., 2017)
*Terminalia arjuna* (Roxb. ex DC.) Wight & Arn. leaf	Gold nanoparticles (TA-AuNPs)	TA-AuNPs induces the mitotic cell division and pollen germination. TA-AuNPs showed a non-cytotoxic effect on *Allium cepa* root tip cells and *Gloriosa superba* pollen grains.	(Gopinath et al., 2013)
*Terminalia bellirica* (Gaertn.) Roxb. fruit pericarp	Silver nanoparticles (TB-AgNPs)	TB-AgNPs at 120 μg/mL killed 69.1% colon cancer cells and 65.2% breast cancer cells. The TB-AgNPs did not cause cytotoxic effects against normal cardiac and skeletal muscle cells.	(Nampoothiri et al., 2018)
*Terminalia belerica* (Gaertn.) Roxb. fruit	Copper nanoparticles (TM-CuONPs), Iron nanoparticles (TM-FeONPs), Zinc nanoparticles (TM-ZnONPs).	Inhibition zone of TM-CuONPs, TM-FeONPs, and TM-ZnONPs against *Staphylococcus aureus* ranged from 22–24 mM.	(Akhter et al., 2019)
*Terminalia catappa* L. leaf	Silver nanoparticles (TC-AgNPs)	TC-AgNPs showed antibacterial activity against both *S. aureus* and *E. coli*. Nylon cloth-TC-AgNPs exhibited antibacterial activity against the organisms with a strength of 3 to 13 times greater than TC-AgNPs.	(Rohaeti and Rakhmawati, 2017)
*Terminalia chebula* Retz. leaf	Gold nanoparticles (TC-AuNPs)	The antibacterial activity of TC-AuNPs against Gram-positive *S. aureus* was better than Gram-negative *E. coli* measured by the well diffusion method.	(Mohan Kumar et al., 2012)
*Terminalia mantaly* H. Perrier	Silver nanoparticles (TM-AgNPs)	MIC values of the TM-AgNPs (3.12 µg/mL) were 40 times lower than those of the extract (125 µg/mL).	(Majoumouo et al., 2019)
*Terminali arjuna* (Roxb. ex DC.) Wight & Arn. bark extract	Gold nanoparticles (AuNPs)	Co-administration with green synthesized AuNPs with size ranging between 20 and 40 nM along with acetaminophen showed effective significant recovery in the hematological alterations of male Wistar rats.	(Mitra et al., 2019)
*Terminalia arjuna* (Roxb. ex DC.) Wight & Arn. bark extract	Gold nanoparticles (TA-PdNPs)	The TA-PdNPs were utilized as an efficient catalyst for Heck and Suzuki type C-C coupling reactions and degradation of organic dyes in aqueous medium making it useful in synthetic organic chemistry and the removal of toxic industrial pollutants, respectively.	(Garai et al., 2018)
*Terminalia arjuna* (Roxb. ex DC.) Wight & Arn. bark extract	Silver nanoparticles (AgNPs)	AgNPs were spherical in shape ranges with 40–50 nM in size. These nanoparticles showed the inhibition of *Staphylococcus aureus* and *Pseudomonas aeruginosa* bacteria.	(Koparde and Gaikwad, 2007)
*Terminalia bellirica* (Gaertn.) Roxb. fruit extract	Gold nanoparticles (AuNPs)	AuNPs were spherical shape ranges with 20–30 nM and found to be effective against *Candida tropicalis* and *Candida albicans* isolated from clinical samples. AuNPs also effectively worked as free radical scavenging activity.	(Annavaram et al., 2017)
*Termanilia arjuna* (Roxb. ex DC.) Wight & Arn. bark extract	Metal oxide nanoparticles: Copper nanoparticles (CuNPs) and Zinc nanoparticles (ZnNPs)	CuNPs exhibited maximum antibacterial efficacy than ZnNPs against the entire organism tested. *K. pneumoniae* showed high resistance to both the biosynthesized nanoparticles. CuNPs exhibited maximum efficacy when compared to ZNPs in antihemolytic activity against hypotonic and heat-induced hemolysis of erythrocytes.	(Anuradha et al., 2017)
*Terminalia arjuna* (Roxb. ex DC.) Wight & Arn. leaf extracts	Gold nanoparticles (AuNPs)	AuNPs were treated with two different concentrations (500 and 1,000 μM) of *Gloriosa superba* seeds. Au NPs exposure at 1,000 μM concentration has the most significant effect on seed germination rate and vegetative growth of *G. superba*. This is the first report on Au NPs as a biocompatibility material to enhance the seed yield of this endangered medicinal plant.	(Gopinath et al., 2013)
*Terminalia arjuna* (Roxb. ex DC.) Wight & Arn. bark extract	Copper nanoparticles (CuNPs)	The *in vitro* antimicrobial activity was found to be effective for CuNPs dried at room temperature when compared to CuNPs dried at 70°C. From this study, CuNPs shows a very good antioxidant property.	(Yallappa et al., 2013)
*Terminalia catappa* L. leaf extract	Gold nanoparticles (AuNPs)	*Terminalia catappa* L. (TC) leaf extract was treated with chloroauric acid solutions, showing a rapid reduction of chloroaurate ions leading to the formation of highly stable AuNPs in solution. AuNPs (10–35 nM size; average size 21.9 nM) can be used as the reducing and stabilizing agent.	(Ankamwar, 2010)

## Possible Mechanism of Action of Extracts of *Terminalia* sp. for Various Pharmacological Activities

**Figure 5:**
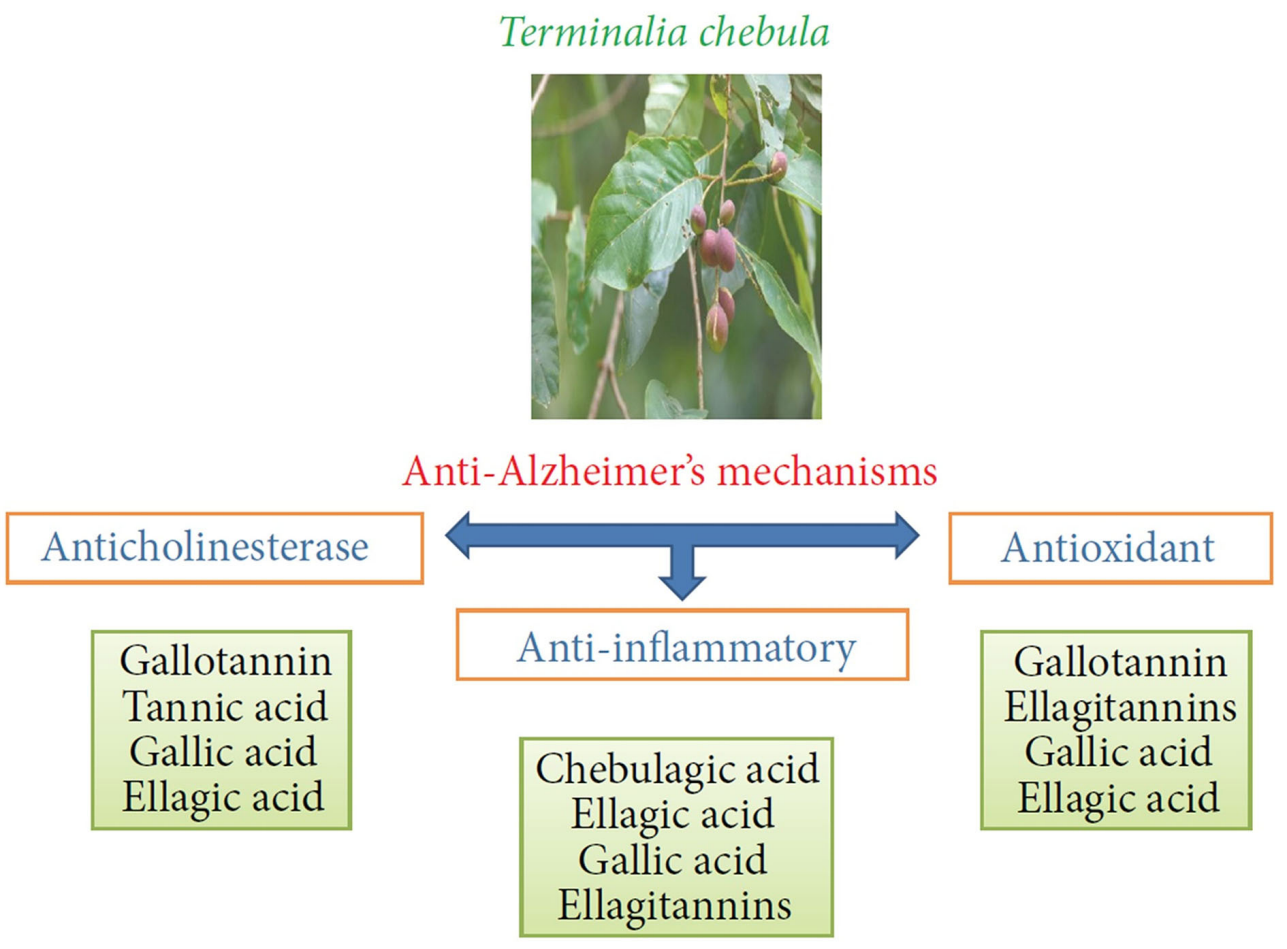
Potential mechanism of anti Alzheimer’s therapy through the anticholinesterase, antiinflammatory, and antioxidant properties of *T. chebula* Retz. Reproduced under the Creative Commons Attribution License (Afshari et al., 2016).

Triphala, a common Ayurvedic formulation which consists of the powder of 3 plants of *Terminalia* sp. such as *Phyllanthus emblica* L.*, Terminalia chebula* Retz. and *Terminalia bellirica* (Gaertn.) Roxb., has been used for long times in the traditional system of medicine for the treatment and prevention of ailments that worry the aging population, and its preclinical studies have confirmed most of its ethnomedicinal claims which are mediated by the myriad biochemical mechanisms (Baliga et al., 2015). In 2002, Saleem et al. (2002) have investigated the cytotoxicity potential of fruit extracts of *T. chebula* Retz., which according to him can decrease the number of cells in immortalized and cancer cell lines by preventing the proliferation rate of the cell and by inducing the cell death. His group stated that at lower concentration, the extract was able to induce the cellular pathways that resulted in the apoptosis process, whereas at the higher concentrations, the extract showed direct toxic effects, resulting in the rapid necrotic cell death (Saleem et al., 2002). Leaf extracts of *Terminalia muelleri* Benth. was reported to show inhibitory potential against the *Staphylococcus aureus* and the authors also stated that the leaf extract induced the shrinkage and thinning of the cell wall mechanism resulting in the inhibition activity (Anam et al., 2010; Cock, 2015). *T. paniculata* Roth extracts have been reported to alter the levels of biomarkers of hepatotoxicity *in vivo*, indicating a hepatoprotective activity. The extracts of *T. paniculata* Roth have been reported to exhibit antioxidant and hepatoprotective activities by altering the levels of biomarkers of hepatotoxicity under *in vivo* condition, thus protecting the liver by blocking the lipid peroxidation process which could damage the internal tissues (Eesha et al., 2011; Cock, 2015). Alzheimer’s disease is considered debilitating dementia, and only a few therapeutic possibilities are presently existing to alter the expressions of the disease and among them, the extracts of *T. chebula* Retz. has been documented to possesses pharmacological activities pertinent to the dementia treatment. The possible anti alzheimer’s desease mechanism of action through the anticholinesterase, antiinflammatory, and antioxidant properties of *T. chebula* Retz. has been propose (Afshari et al., 2016) (**Figure 5**).

In a study, Yeh et al. (2014) have demonstrated that the *Terminalia catappa* L. leaf extract exhibited an inhibitory effect on several vital steps of metastasis, that includes invasion and migration of cell, by regulating the actions and protein level of urokinase-type plasminogen activator and its natural inhibitor. They also showed that the plant extract could be able to effectively inhibit the phosphorylation of the ERK1/2 signaling pathways by the downregulation of the transcription factors SP-1 and NF-κB DNA binding activities, that leads to the suppression of urokinase-type plasminogen activator and inhibition of metastasis (Yeh et al., 2014). In another study, Pinheiro Silva et al. (2015), showed that the aqueous fraction from the leaves of *Terminalia catappa* L. possesses anti *Helicobacter pylori* activity and excellent preventive and curative activity on the acute and chronically induced gastric ulcers. The detailed mechanism of action associated in the gastro-protection are connected to the nitric oxide pathway, an increase in the mucus level and the endogenous prostaglandins, and this fraction was able to cure the ulcers through the inhibition of the matrix metallo proteinase activities (MMP-2 and MMP-9) (Pinheiro Silva et al., 2015).

## Preclinical and Clinical Studies on Active Compounds From *Terminalia* sp.

### Preclinical Studies

There are currently few preclinical and clinical studies of *Terminalia* species, as far as our literature research. Out of the few reports, Ekambaram et al. (2018), evaluated the acute toxicity of *T. chebula* Retz. fruit hydrolyzable tannin-rich methanolic extract, which did not exhibit any toxicity even at a dose administration of 5000 mg/kg/p.o. for 14 days. Thus, *Terminalia chebula* Retz. fruit extracts can be considered as safe. HPLC analysis showed the presence of (53) chebulagic acid, (5) corilagin, and (9) chebulinic acid. On the other hand, *Terminalia* sp. extracts significantly affected some biochemical parameters in both male and female rats, which cause a reduction in body weight. This effect could be attributed to the relationship between high tannin content in *T. chebula* Retz. extracts and their influence on decreased feed intake, growth rate, feed efficiency, net metabolizable energy, and protein digestibility (Ekambaram et al., 2018).

Similarly, Awotunde et al. (2019), evaluated the subacute toxicity of *Terminalia schimperiana* Hochst. ex Engl. & Diels (synonym of *Terminalia glaucescens* Planch. ex Benth.), water extracts in male rats. It was reported that extracts did not have a toxic effect in any organs at doses of 1000, 2000, and 3000 mg/kg body weight, nor had any effect on the biochemical parameters of treated rats. Also, Das et al. (2015), reported the acute and subacute toxicity of methanol extracts of *Terminalia citrina* (Gaertn.) Roxb. leaves in female Sprague rats at doses of 250, 500, and 1000 mg/kg bodyweight for 28 days. *Terminalia* sp. treatment had no significant effect on biochemical parameters like alanine aminotransferase, aspartate aminotransferase, alkaline phosphatase, glucose, and creatinine, which indicates no detriment of liver and kidney functions. Also, *T. citrina* (Gaertn.) Roxb. extracts did not affect hematological parameters such as white blood count, red blood cell count, platelet count and hemoglobin content, neutrophil, lymphocyte, monocyte, eosinophil, and hematocrit content (Das et al., 2015).

### Clinical Studies

Clinical studies regarding the potential bioactivities of *Terminalia* species are scarce, which limits its potential use as a biopharmaceutical agent against several diseases. Here we briefly summarize the available reports. C. U. Kumar et al. (2015), performed a randomized, double-blind, placebo-controlled, cross over study to evaluate the analgesic activity and safety of a single oral administration of *Terminalia chebula* Retz. using a hot air pain model in 14 healthy human participants (18–45 years old). The authors administered a single dose of two capsules of 500 mg of an aqueous extract of fruits of *Terminalia chebula* Retz., which contained no less than 15% (9) chebulinic acid, 10% of (53) chebulagic acid and not less than 15% of other low molecular weight hydrolyzable tannins. *T. chebula* Retz. increased the mean percentage change of pain threshold time and pain tolerance time compared to placebo treatments. This effect was mainly attributed to the anti-inflammatory activity of (3) gallic acid, (4) ellagic acid, and (5) corilagin from extracts of fruits of *T. chebula* Retz. and the anti-arthritic effect of the hydrolyzable tannins of *Terminalia* sp.

Three studies evaluated the effect of *Terminalia arjuna* (Roxb. ex DC.) Wight & Arn. in patients with cardiovascular diseases (Kapoor et al., 2015; Maulik et al., 2016; Priya et al., 2019). The report of Priya et al. (2019), found that systolic and diastolic blood pressure decreased after 1 month of *T. arjuna* (Roxb. ex DC.) Wight & Arn. therapy, which consisted of the oral administration of 3 g of a mixture of *T. arjuna* (Roxb. ex DC.) Wight & Arn. mixed in 250 mL of boiled milk twice daily. The bioactive effect was attributed to the presence of adrenergic β2 receptor agonistic action. This may be partially related to the antioxidant activity of *T. arjuna* (Roxb. ex DC.) Wight & Arn., possibly related to the protection of myocardial ischemic reperfusion injury. *Terminalia arjuna* (Roxb. ex DC.) Wight & Arn. treatment also decreased total cholesterol levels (235.02 to 210.80 mg/dL) and serum LDL levels (134.40 to 121.5 mg/dL). The second study was conducted as a double-blind, parallel, randomized, placebo-controlled add-on clinical trial by Maulik et al. (2016) to assess the safety of a standardized water extract of stem bark of *T. arjuna* (Roxb. ex DC.) Wight & Arn. in chronic heart failure patients.

Nevertheless, the *T. arjuna* (Roxb. ex DC.) Wight & Arn. treatment, even when it was well-tolerated, did not change the left ventricular ejection fraction or secondary outcome measures. The third report evaluated the cardioprotective effect of *T. arjuna* (Roxb. ex DC.) Wight & Arn. on classical and immuno-inflammatory markers in coronary artery disease by administering 500 mg twice a day to eight patients. *Terminalia* sp. treatment significantly down-regulated the triglycerides, VLDL-C, and immune-inflammatory markers in stable coronary artery disease after 3 months, and the effect was maintained after 6 months with decreased total cholesterol levels.

## Concluding Remarks and Perspectives

Extracts from plants of the genus *Terminalia* sp. are a rich source of phytochemicals such as terpenes, flavonoids, and phenolic acids. It is suggested that these molecules are related to the antibacterial, antioxidant, antiinflammatory, antifungal, antiviral, antiparasitic, antidiabetic, and anticancer activity of *Terminalia* plants. Several reports have associated the ethnopharmacological potential of plant extracts or phytochemicals isolated from medicinal plants and plant foods with their bioavailability. However, as far as our literature research, we did not find bioavailability reports as well as any pharmacokinetic data. Thus, it is unknown if these molecules will exert any bioactivity in humans.

Essentially, *Terminalia* plants are yet to be actively explored on the molecular and docking scales, in this case, further exploration of the mechanisms involved in their enzymes modulation and radical scavenging abilities is worth considering. Also, there is little clinical research on the bioactivity of *Terminalia* species, which practically limits their potential use as a pharmaceutical against diseases. More preclinical and clinical studies are needed if extracts or isolated compounds from *Terminalia* species want to be used as biopharmaceutical agents. There is still a need for pharmacokinetic and toxicological studies to be able to determine if *Terminalia* sp. is suitable for the development of a drug or herbal-based remedy; there is also a huge lack of studies regarding the effective doses of *Terminalia* sp. for prevention/treatment of the pathologies mentioned above. Also, there is an important flaw in the diversity of studies presented here, as the reports lack reproducibility because bioactivity studies use different concentrations of fruit, bark, and leaves *Terminalia* sp. extracts, nor they use the same plant parts.

## Author Contributions

JKP and GD conceptualized the whole concept. JKP, GD, D-YK, CF, EG-G, JH, VN, WM, MP, MN, AS, and RN wrote, reviewed, and edited the manuscript. JKP, HS-S, and BS helped in the collection of literature, review, and editing of the manuscript. All authors contributed to the article and approved the submitted version.

## Funding

This work was supported by Korea Institute of Planning and Evaluation for Technology in Food, Agriculture and Forestry (IPET) through Innovative Food Product and Natural Food Materials Development Program (No.319049-3), funded by Ministry of Agriculture, Food and Rural Affairs (MAFRA).

## Conflict of Interest

The authors declare that the research was conducted in the absence of any commercial or financial relationships that could be construed as a potential conflict of interest.
