# Supplementary material for: Plants of the Genus Terminalia: An Insight on Its Biological Potentials, Pre-Clinical and Clinical Studies
Source: Front Pharmacol. 2020 Oct 8;11:561248. doi: 10.3389/fphar.2020.561248 (PMC7578430; doi:10.3389/fphar.2020.561248)
Supplement: Supplementary file 1 [file Table_1.docx]

**Supplementary Table 1: Detailed taxonomic names of the *Terminalia* species used in the review article**

|  | **Plant common name** | **Plant full scientific name MANDATORY DATABASE Kew MPNS; www.theplantlist.org; www.plantsoftheworldonline.org** |
| --- | --- | --- |
| 1 | Billy Goat Plum | *Terminalia ferdinandiana* Exell |
| 2 | Aadalu, adityalu, ahaliva, ahdan koddai, akkantam, aksa | *Terminalia bellirica* (Gaertn.) Roxb*.* |
| 3 | a-ru, a-ru-ra, abhaya | *Terminalia chebula* Retz. |
| 4 | Arccunam, arjon, arjun, arjuna, bilimatti | *Terminalia arjuna* (Roxb. ex DC.) Wight & Arn. |
| 5 | Darot, sabaranya, subagh, olbugoi, olbukoi | *Terminalia brownii* Fresen. |
| 6 | NA | *Terminalia argentea* Mart. |
| 7 | Abrofo nkatie, almendro de la india, almenron | *Terminalia catappa* L. |
| 8 |  | *Terminalia coriacea* (Roxb.) Wight & Arn. (*Terminalia coriacea* Spreng.) |
| 9 | Balak, honguigon | *Terminalia laxiflora* Engl. |
| 10 | A-ntud, apela, dagnela | *Terminalia macroptera* Guill. & Perr. |
| 11 | Gab, amangwe | *Terminalia sericea* Burch. ex DC*.* |
| 12 | NA | *Terminalia schimperiana* Hochst. ex Engl. & Diels ((synonym of *Terminalia glaucescens* Planch. ex Benth.) |
| 13 | NA | *Terminalia grandiflora* Benth. |
| 14 | NA | *Terminalia carpentariae* C.T.White (synonym of *Terminalia hadleyana* subsp. *carpentariae* (C.T.White) Pedley) |
| 15 | Vanmaruthu | *Terminalia paniculata* Roth |
| 16 | Asan, indian laurel | *Terminalia alata* Roth |
| 17 | NA | *Terminalia corticosa* Pierre ex Laness |
| 18 | NA | *Terminalia glaucescens* Planch. ex Benth. |
| 19 | Australian almond | *Terminalia muelleri* Benth. |
| 20 | Lebombo cluster-leaf, Lebombotrosblaar (Afr.), amaNgwe-amnyama, amaNgwe-omphofu (isiZulu), mambonjwane (SiSwati) | *Terminalia phanerophlebia* Engl. & Diels |
| 21 | Shinglewood, English afara (from Yoruba); white afara; ofram (from Asanti, Twi); shingle wood; yellow pine; white mukonja (W Cameroons); Congo walnut. French franké (from Anyi); limba (ex Congo); limbo (ex Gabon); limbo blanc, or limbo clair (with light-coloured wood); limbo noir (with dark-coloured heart-wood); noyer du moyambe (i.e. Congo walnut, with dark-coloured heart-wood). | *Terminalia superba* Engl. & Diels |
| 22 | English black afara (Chalk & al., Kunkel); black bark (Dalziel, Kunkel, Irvine); black barked terminalia (Chalk & al.,); brimstone wood (Chalk & al.); satinwood (Chalk & al., Dalziel, Kunkel); shingle wood (Dalziel, Irvine, Kunkel); yellow terminalia (Chalk & al., Dalziel, Irvine); yellow pine (Kunkel). French bois satiné (Kerharo & Boquet); framiré (auctt); | *Terminalia ivorensis* A.Chev. |
| 23 | Madagascar Almond, Umbrella Tree | *Terminalia mantaly* H. Perrier |
| 24 | NA | *Terminalia albida* Scott-Elliot |
| 25 | NA | *Terminalia avicennioides* Guill. & Perr*.* |
| 26 | Aagra, aamla, aaunlesa, amalaki, amrtaphala | *Phyllanthus emblica* L. |
| 27 | Myrobalan | *Terminalia citrina* (Gaertn.) Roxb. |

NA= Not available
